# Ginsenosides Improve Nonalcoholic Fatty Liver Disease *via* Integrated Regulation of Gut Microbiota, Inflammation and Energy Homeostasis

**DOI:** 10.3389/fphar.2021.622841

**Published:** 2021-02-12

**Authors:** Wenyi Liang, Kun Zhou, Ping Jian, Zihao Chang, Qiunan Zhang, Yuqi Liu, Shuiming Xiao, Lanzhen Zhang

**Affiliations:** ^1^ School of Traditional Chinese Medicine, Beijing University of Chinese Medicine, Beijing, China; ^2^ Institute of Chinese Materia Medica, China Academy of Chinese Medical Sciences, Beijing, China

**Keywords:** ginsenosides, nonalcoholic fatty liver disease, network pharmacology, gut microbiota, inflammation

## Abstract

Ginseng, the root and rhizome of *Panax ginseng* C. A. Mey., is a famous herbal medicine, and its major ginsenosides exert beneficial effects on nonalcoholic fatty liver disease (NAFLD). Due to the multicomponent and multitarget features of ginsenosides, their detailed mechanisms remain unclear. This study aimed to explore the role of ginsenosides on NAFLD and the potential mechanisms mediated by the gut microbiota and related molecular processes. C57BL/6J mice were fed a high-fat diet (HFD) supplemented or not supplemented with ginsenoside extract (GE) for 12 weeks. A strategy that integrates bacterial gene sequencing, serum pharmacochemistry and network pharmacology was applied. The results showed that GE significantly alleviated HFD-induced NAFLD symptoms in a dose-dependent manner. Furthermore, GE treatment modulated the HFD-induced imbalance in the gut microbiota and alleviated dysbiosis-mediated gut leakage and metabolic endotoxemia. Additionally, 20 components were identified in the mouse plasma after the oral administration of GE, and they interacted with 82 NAFLD-related targets. A network analysis revealed that anti-inflammatory effects and regulation of the metabolic balance might be responsible for the effects of GE on NAFLD. A validation experiment was then conducted, and the results suggested that GE suppressed NF-κB/IκB signaling activation and decreased the release and mRNA levels of proinflammatory factors (TNF-α, IL-1β and IL-6). Additionally, GE promoted hepatic lipolytic genes (CPT-1a), inhibited lipogenic genes (SREBP-1c, FAS, ACC-1) and improved leptin resistance. These findings imply that the benefits of GE are involved in modulating the gut microbiota, enhancing the gut barrier function, restoring the energy balance, and alleviating metabolic inflammation. Moreover, GE might serve as a potential agent for the prevention of NAFLD through the integration of prebiotic, anti-inflammatory and energy-regulatory effects.

## Introduction

Nonalcoholic fatty liver disease (NAFLD), which is a chronic and multifactorial liver disease, is becoming a growing health problem worldwide (Younossi et al., 2016). At early stages, the disease manifests as simple hepatic steatosis, which is frequently observed in patients with obesity, dyslipidemia, and diabetes (Polyzos et al., 2019). During its progression, the disease gradually evolves into a broad spectrum of liver diseases, including nonalcoholic steatohepatitis, liver fibrosis, cirrhosis, and even hepatocellular carcinoma (Friedman et al., 2018). Currently, a widely accepted hypothesis regarding multiple parallel hits explains NAFLD pathogenesis, including insulin resistance, hepatic lipid accumulation, inflammatory reaction, oxidative stress, gut microbiota, and genetic predisposition (Friedman et al., 2018; Kolodziejczyk et al., 2019). The existing therapeutic strategy only aims to control the progression of obesity, diabetes, and dyslipidemia by adjusting lifestyle factors, increasing insulin sensitivity, reducing inflammation, and protecting liver cells and thus does not yield ideal therapeutic effects. Despite urgent medical needs, the FDA has not yet approved any drugs for NAFLD therapy. Interestingly, natural extracts and phytochemicals have gradually attracted attention due to their extraordinary hepatoprotective abilities (Cremonini et al., 2018; Tang et al., 2018; Li et al., 2019).

Ginseng, the root and rhizome of *Panax ginseng* C. A. Mey., is used as a traditional Chinese medicine and an adaptogen for thousands of years to ameliorate weakness and fatigue, prolong life, improve emotional well-being, and tonify body (Jia and Zhao, 2009; Patel and Rauf, 2017). Ginsenosides are the major active components, and various biological functions were recognized, such as effects against obesity, hyperglycemia and NAFLD (Hwang et al., 2018; Li and Ji, 2018; Xu et al., 2020; Zhang et al., 2020). However, the above-described research mainly focused on the activities and mechanisms of a single component, and the synergetic and comprehensive effects of multiple components remain unclear. Therefore, this study explores the benefits of ginsenoside extract (GE) on NAFLD and its potential integrated mechanisms.

It is well known that most herbal medicines are orally administered. Phytochemicals, such as ginsenosides, are usually metabolized by the gut microbiota and then transported into the bloodstream (Xu et al., 2017). During their processing, the unabsorbed compositions remaining in the intestine might affect the intestinal microbiota, whereas others entering the blood selectively interact with multiple targets, which might be synergetic and advantageous for the amelioration of pathological conditions (Zhao et al., 2015). However, the multicomponent, multitarget, and multichannel features of herbal medicines hinder the interpretation of their mechanisms. High-throughput sequencing has recently been used to analyze the profile characteristics and functions of disease-related microbial communities (Wu et al., 2019). In addition, network pharmacology has been proposed as a new bioinformatics strategy for investigating the interaction between components and biology by mapping drug-target-disease networks. Increasing lines of evidence suggest that the above-mentioned tools are powerful for improving the understanding of the therapeutic mechanisms of herbal medicines for complicated diseases (Huang et al., 2019; Mao et al., 2019).

By considering multiple ginsenosides and their crosstalk with organisms, we intend to use an integrated strategy that combines bacterial gene sequencing, serum pharmacochemistry and network pharmacology to explore the multiscale mechanisms of GE against NAFLD.

## Materials and Methods

### Plant Extract

GE was prepared and provided by Changchun University of Chinese Medicine, Changchun, Jilin, China, and stored at 4 °C until use. The preparation, qualitative and quantitative analyses of GE were performed as detailed in the Supplementary Information. Ultra-performance liquid chromatography/tandem mass spectrometry (UPLC-MS^n^) analysis revealed the complex chemical profile of GE, and a total of 56 components were identified. In addition, the contents of 9 main ginsenosides in GE were listed in Table 1.

**TABLE 1:** The contents of nine main ginsenosides in GE (*n* = 3).

Analyte	Content (%)	RSD (%)
Re	14.46	1.62
Rg1	3.49	0.24
Rf	2.02	0.16
Rb1	12.78	1.60
Rc	13.02	1.54
20(*S*)-Rg2	1.00	0.22
Rb2	7.52	0.85
20(*S*)-Rh1	0.27	0.54
Rd	3.64	0.48

### Animal Studies

#### Animal Groups and Handling

The animal studies were approved prior to their implementation by the Animal Care Committee of Beijing University of Chinese Medicine (Approval No. 2018–3,031). Six-week-old C57BL/6J male mice were raised under SPF conditions (12-h light/12-h dark cycle at 22 ± 2 °C and 60 ± 5% relative humidity) and had free access to their diet and water. After an adaptive period of 1 week, the mice were randomly divided into four groups: 1) the ND group was administered a normal chow diet (10% calories from fat); 2) the HFD group was administered a high-fat diet (60% calories from fat); 3) the GEL group was administered the HFD supplemented with 100 mg GE/kg body weight; and 4) the GEH group was administered the HFD supplemented with 200 mg GE/kg body weight. The experiment lasted for a total of 12 weeks. The body weight was measured once a week, and the food intake and waste were measured daily. Fresh fecal samples were collected at the end of the trial. The mice were then anesthetized with isoflurane, and samples of serum, liver, perinephric adipose tissues (PATs), epididymal adipose tissues (EATs), subcutaneous adipose tissues (SATs) and ileum were collected and stored at −80 °C.

#### Biochemical Analysis

The levels of serum total cholesterol (TC), triglyceride (TG), low-density lipoprotein cholesterol (LDL-C), high-density lipoprotein cholesterol (HDL-C), aspartate aminotransferase (AST), alanine aminotransferase (ALT) and hepatic TC and TG were measured using commercial kits according to the provided instructions (Jiancheng Bioengineering Institute, Nanjing, China).

#### Histopathology

Livers and EATs were fixed in 4% paraformaldehyde for 24 h. The fixed liver tissues and EATs were embedded in paraffin blocks and sectioned into 3-μm-thick sections. The sections were deparaffinized for hematoxylin-eosin (H&E) staining. Images were acquired using a Nikon ECLIPSE Ts2R microscope.

### Gut Microbiota Analysis

Microbial DNA was extracted from the fecal samples using the E. Z.N.A. Soil DNA Kit. Its concentration and purification were determined using a NanoDrop 2000 (Thermo, Wilmington, DE, USA), and its quality was checked by 1% agarose gel electrophoresis. The fecal DNA was then subjected to 16S rRNA sequencing as described previously (Ma et al., 2019). The sequencing data was deposited in the SRA database at the NCBI (https://www.ncbi.nlm.nih.gov/sra) under accession number PRJNA673766. For bioinformatics analysis, alpha-diversity analyses of the mouse fecal microbiota, including analysis of the Shannon index and rarefaction curve analysis at the operational taxonomic unit (OTU) level, were performed using mothur software (Schloss et al., 2009). A beta-diversity analysis was conducted to explore the similarities or differences in the community composition between different grouped samples by calculating the unweighted UniFrac distance matrices. A plot of the principal coordinate analysis (PCoA) based on the OTU abundance was generated and visualized with R software. The linear discriminant analysis (LDA) effect size (LEfSe) (Segata et al., 2011) was used for the nonparametric factorial Kruskal-Wallis sum-rank test followed by LDA analysis to identify the altered bacterial taxa at the OTU level among the different treatment groups, and two filters (*p* <0.05 and LDA score >3) were applied to the present features. A variance inflation factor (VIF) analysis was performed to screen out environmental factors (NAFLD-related parameters) with low multicollinearity and VIF values >10. Spearman’s correlation analysis was conducted to calculate the correlations between bacteria and disease parameters, and the results were visualized in a heatmap using R software.

### Identification of the Components of GE in the Plasma

#### Sample Preparation

The mice received an oral dose of 200 mg GE/kg body weight. Blood samples were collected from the heart into heparin tubes after GE administration. Plasma was obtained after centrifugation at 3,500 rpm and 4 °C for 10 min. MeOH (800 µL) was added to 200 µL of plasma, and the mixture was centrifuged at 14,000 rpm and 4 °C for 10 min. The supernatant was then dried under a steady stream of nitrogen gas at 35 °C, and the residues were dissolved in 100 µL of 70% methanol for UPLC-MS^n^ analysis.

#### UPLC-MS^n^ Conditions

An electron spray ionization hybrid linear ion trap quadrupole-Orbitrap mass spectrometer coupled with a Thermo Accela 600 HPLC system (Thermo Scientific, Bremen, Germany) was used for serum pharmacochemistry analysis. The ingredients were separated with an ACQUITY HSS T3 C18 UPLC column (100 × 2.1 mm i. d., 1.8 µm) at 30 °C. Acetonitrile (A) and 0.1% (*v/v*) formic acid in water (B) were used as the solvents for elution at a flow rate of 0.3 ml/min, and the gradient program was as follows: 0–1 min, 20%; 1–10 min, 20–35% A; 10–22 min, 35–70% A; 22–24 min, 70–90% A; 24–26 min, 90% A; 26–27 min, 90–20% A; and 27–30 min, 20% A. The injection volume was 2 µL. Mass spectra were acquired in the negative ionization mode with a scan range of *m/z* 200–1,500. The instrument was operated under the following setting parameters: capillary voltage, −35 V; source voltage, 3 kV; tube lens voltage, −110 V; capillary temperature, 350 °C; sheath gas (nitrogen) flow, 40 arb., and auxiliary gas (nitrogen) flow, 20 arb. Xcalibur 3.0 software was used for tentative peak identification.

### Network Pharmacology Study

The therapeutic targets of NAFLD were mined from the GeneCards (https://www.genecards.org/) and Integrative Pharmacology-based Research Platform of Traditional Chinese Medicine (TCMIP, http://www.tcmip.cn/TCMIP/index.php/Home/) databases (Xu et al., 2019). The *in vivo* targets of the identified chemicals were acquired from several free online databases, including the Swiss Target Prediction database (http://www.swisstargetprediction.ch/) and the PharmMapper database (http://www.lilab-ecust.cn/pharmmapper/submitfile.html). The structural files were imported into the above-mentioned databases to screen the corresponding candidate targets. The disease and drug targets were then mapped to identify the common targets, which were considered the putative targets of GE treatment. To explain the interactions between putative targets, a protein-protein interaction (PPI) network was generated with the STRING platform (https://string-db.org), in which high-confidence data >0.7 were included to ensure the reliability of the analysis. Subsequently, a “drug-component-disease-target” network was constructed and visualized using Cytoscape 3.0.2 (Boston, MA, United States). To elucidate the function of components and their roles in signal transduction, the enriched Gene Ontology (GO) functions and Kyoto Encyclopedia of Gene and Genomics (KEGG) pathways were identified using Bioconductor R software. The enriched KEGG terms were preserved and analyzed based on *p*-value correction <0.05 (Bonferroni step down). The results were visualized and displayed using R software.

### Enzyme Linked Immunosorbent Assay (ELISA)

The levels of tumor necrosis factor-α (TNF-α), interleukin 6 (IL-6), IL-β (all from R&D Systems, MN, United States), LPS (GenScript, NJ, United States), leptin and adiponectin (all from RayBiotech Life, GA, United States) in serum were detected using ELISA kits according to the provided instructions.

### Quantitative Real-Time PCR (qRT-PCR)

RNA from the liver and ileum samples was extracted using the TRIzol reagent (Invitrogen, Carlsbad, CA, United States). Double-stranded DNA was synthesized with a RevertAid First Strand cDNA Synthesis Kit (Thermo, CA, United States). qRT-PCR analyses were performed in triplicate using SYBR Green and the CFX96 Real-Time PCR System (Bio-Rad, Hercules, CA, United States). Glyceraldehyde-3-phosphate dehydrogenase (GAPDH) was used as a housekeeping gene, and the relative expression levels of the analyzed genes were calculated using the 2^-∆Δ*Ct*^ method. The primer sequences are provided in Supplementary Table S1.

### Western Blotting

Liver samples were homogenized in RIPA lysis buffer (Thermo, CA, United States) and protease inhibitor cocktail (Thermo, CA, United States), and total protein was then extracted. The protein samples were separated using a 10% sodium dodecyl sulfate-polyacrylamide gel and transferred onto polyvinylidene fluoride membranes using a Bio-Rad wet transfer unit. The membranes were sealed with 5% skim milk for 1 h at room temperature and incubated overnight at 4 °C with primary antibodies against NF-κB p65, IκBα, p-p65 (Ser 536), *p*-IκBα (Ser 32/36) and *β*-actin (1:1,000; Cell Signaling Technology, MA, United States). The membranes were washed, probed with horseradish peroxidase-conjugated secondary antibody, and detected with an enhanced chemiluminescence detection system (GE ImageQuant). The quantitative analysis was performed by ImageJ 1.51k software.

### Statistical Analysis

All the data are presented as mean ± standard deviation. The data were analyzed by ANOVA with a *post hoc* Tukey test, unpaired Student’s t-test, or nonparametric Mann-Whitney *U* test using SPSS 16.0 software (Chicago, IL, United States). *p* <0.05 was considered statistically significant.

## Results

### GE Intake Attenuates HFD-Induced NAFLD

#### GE Prevents HFD-Induced Abnormal Weight Gain

**FIGURE 1:**
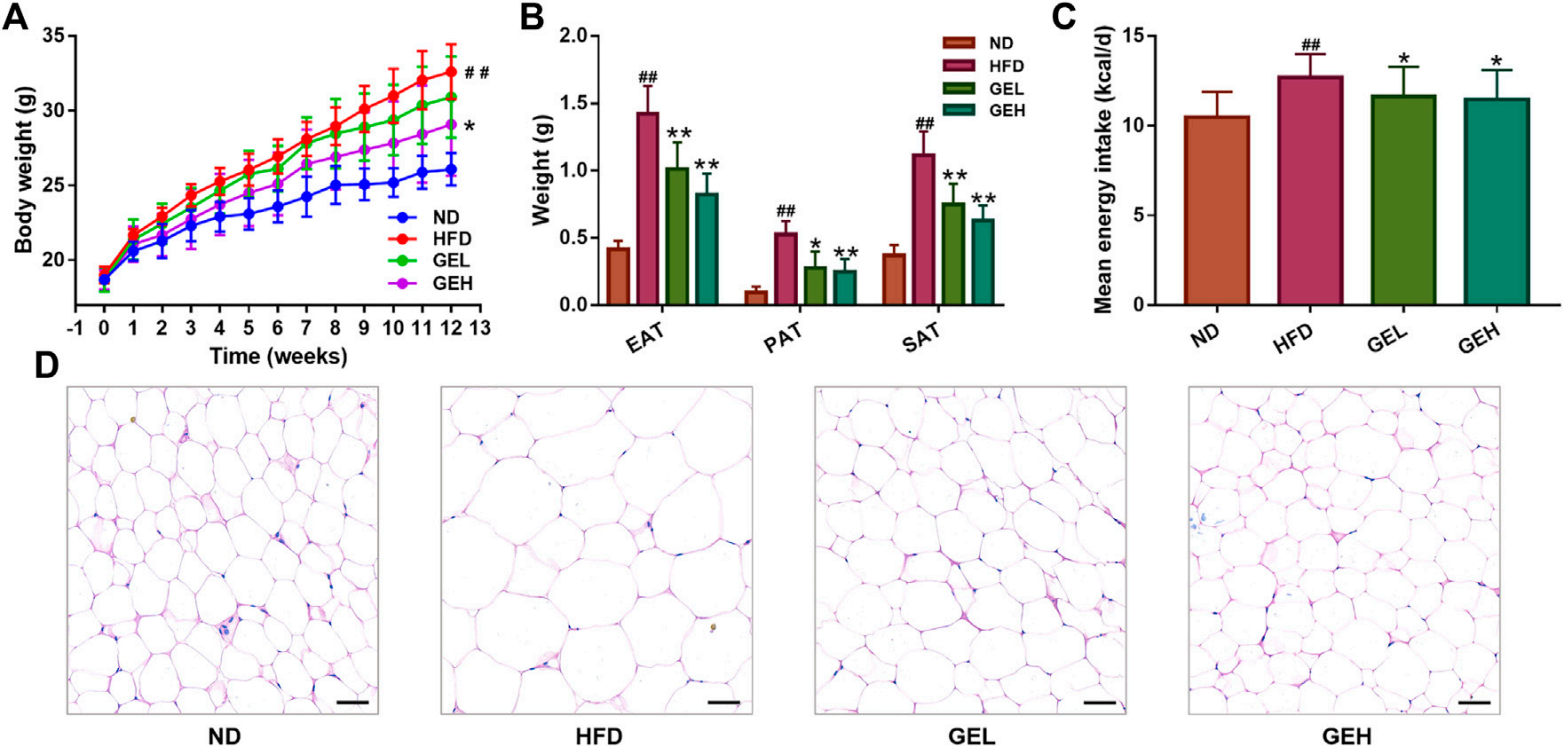
Effects of GE treatment on the abnormal weight gain induced by HFD. **(A)** Body weight. **(B)** White fat tissue weight. **(C)** Energy intake. **(D)** H&E staining of EAT tissues (200× magnification, scale bars = 50 μm). n = 8 per group. ^*#*^ *p* <0.05 or ^*##*^ *p* <0.01 vs. the ND group. *p <0.05 or **p <0.01 vs. the HFD group.

After the 12-weeks intervention, GE treatment prevented the HFD-induced weight gain, particularly the overaccumulation of white fat, including EAT, PAT and SAT (Figures 1A,B). A small reduction in the mean daily caloric intake was detected in the groups that consumed GE (Figure 1C), which suggested that the delayed body weight gain caused by GE might be related to changes in caloric intake to a certain extent. Moreover, GE addition attenuated EAT hypertrophy and increased the number of small adipocytes (Figure 1D).

#### GE Prevents HFD-Induced Hepatocyte Injury and Abnormalities

**FIGURE 2:**
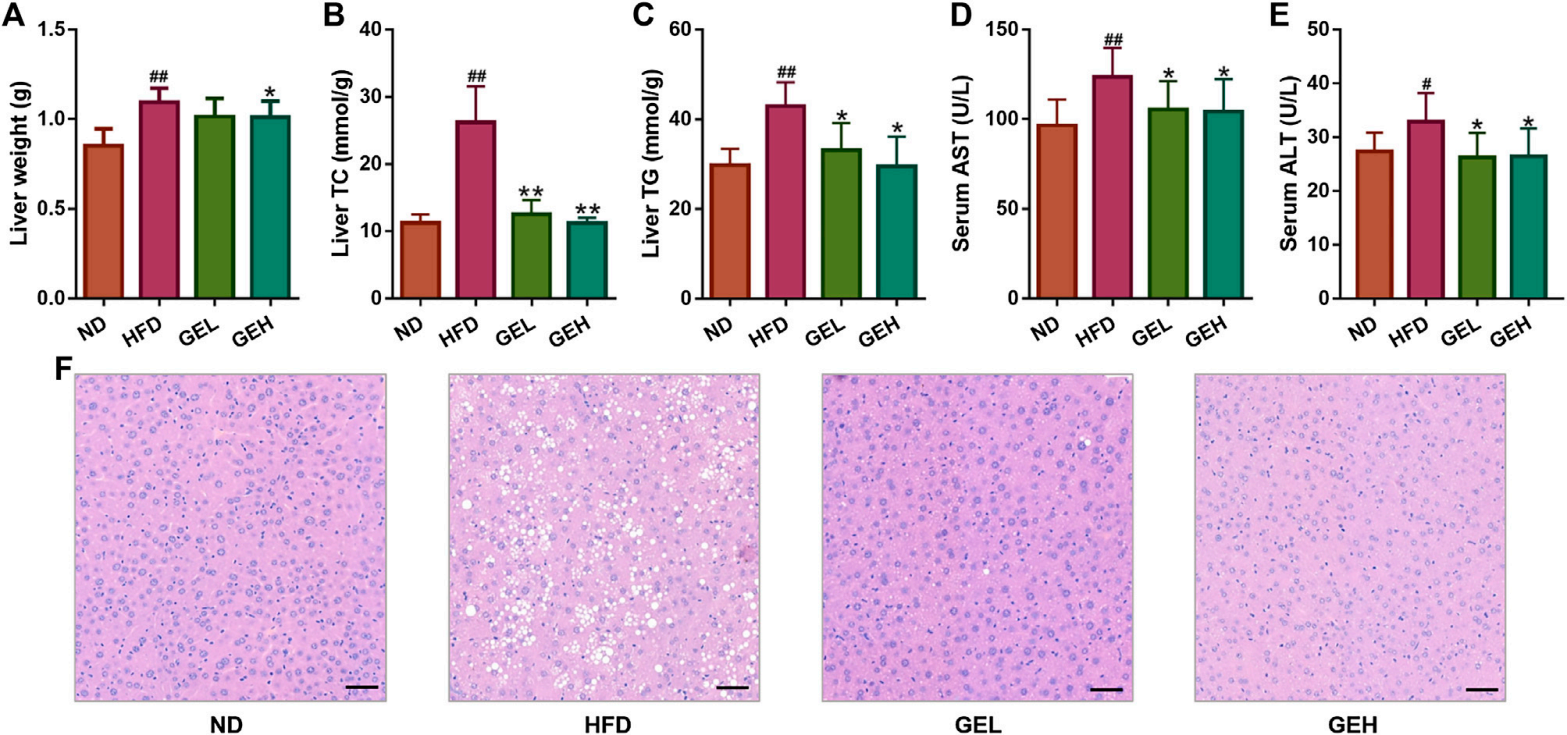
Effects of GE treatment on HFD-induced hepatocyte injury and abnormalities. **(A)** Liver weight. **(B–C)** Hepatic lipid levels. **(D–E)** Serum AST and ALT levels. **(F)** H&E staining of liver tissues (200× magnification, scale bars = 50 μm). n = 8 per group. ^*#*^ *p* <0.05 or ^*##*^ *p* <0.01 vs. the ND group. *p <0.05 or **p <0.01 vs. the HFD group.

Excessive increases in the liver weight induced by HFD were observed in the HFD group. The oral administration of 200 mg/kg GE prevented the abnormal liver weight gain (Figure 2A). Increases in the levels of TC, TG, AST, and ALT were observed in the HFD group, whereas the treatment of the HFD-fed mice with GE lowered these trends in a dose-dependent manner (Figures 2B–E). Furthermore, the liver histopathological analysis clearly showed that the HFD-fed mice exhibited more lipid droplets and obvious macrovesicular steatosis than the ND mice. In contrast, GE intake decreased the hepatic accumulation of lipids (Figure 2F). The above-described results indicated that GE exerts potential hepatoprotective effects and significantly inhibits hepatic steatosis.

#### GE Inhibits HFD-Induced Dyslipidemia

**FIGURE 3:**
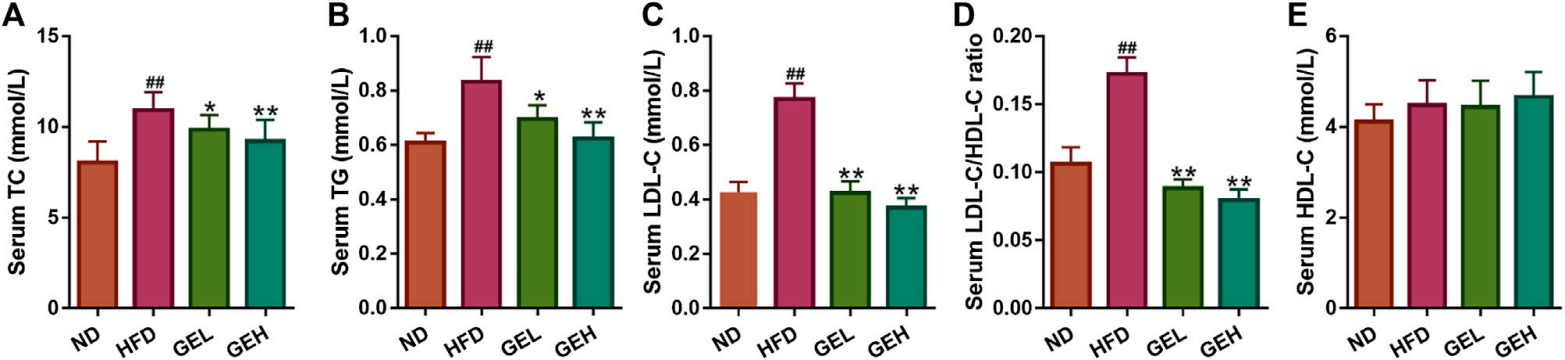
Effects of GE treatment on HFD-induced dyslipidemia. **(A–C)** Serum TC, TG and LDL-C levels. **(D)** Serum LDL-C/HDL-C ratio. **(E)** Serum HDL-C level. n = 8 per group. ^*#*^ *p* <0.05 or ^*##*^ *p* <0.01 vs. the ND group. *p <0.05 or **p <0.01 vs. the HFD group.

The ingestion of the HFD for 12 weeks clearly induced increases in the serum levels of TC, TG, and LDL-C in mice (Figures 3A–C), which suggested that long-term HFD intake caused an aberrant lipid metabolism. Interestingly, the consumption of GE prevented the increases in the levels of serum TC, TG, and LDL-C and the LDL-C/HDL-C ratio in a dose-dependent manner (Figures 3A–D), although no significant difference in the serum HDL-C levels were found among the different groups (Figure 3E). These results indicated the positive role of GE in the regulation of serum lipid metabolism.

### GE Intake Modulates the Gut Microbiota in HFD-Fed Mice

**FIGURE 4:**
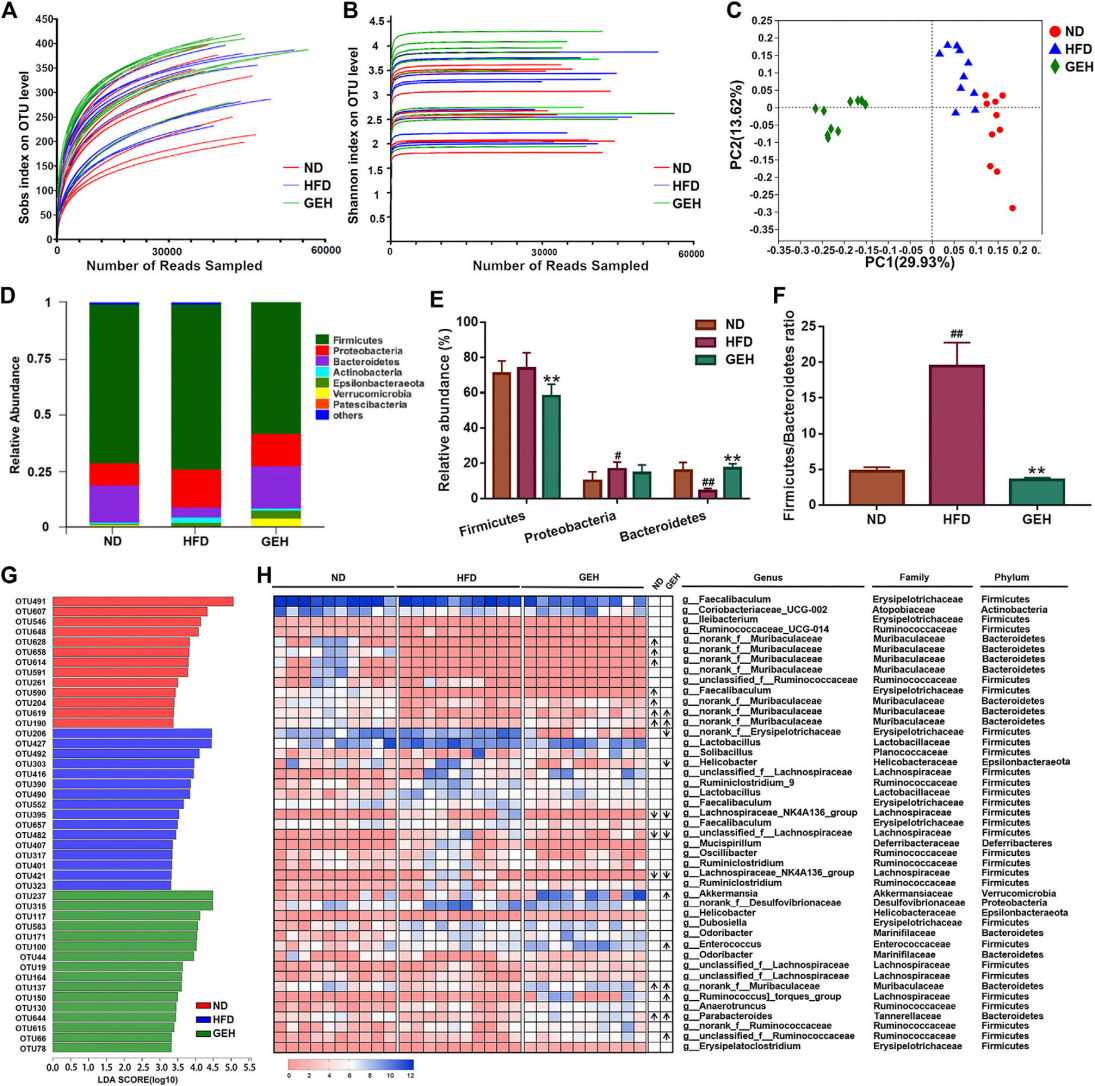
Effects of different treatments on the gut microbial composition. **(A)** Rarefaction curve. **(B)** Shannon curve. **(C)** Unweighted UniFrac-based PCoA plot of the mouse fecal bacterial community. **(D)** Bacterial phylum-level taxonomic profiling. **(E–F)** Relative abundance of dominant phyla and Firmicutes/Bacteroidetes ratio. **(G)** LDA score bar chart generated from the LEfSe analysis (LDA score >3). **(H)** Heatmap showing the abundances of 45 OTUs in different groups based on the LDA. The arrows (“↑” and “↓”) in the boxes represent the relative abundances of OTUs in the ND and GEH groups compared with the HFD group, respectively. n = 10 per group. ^*#*^ *p* <0.05 or ^*##*^ *p* <0.01 vs. the ND group. *p <0.05 or **p <0.01 vs. the HFD group.

The 16S rRNA gene sequencing analysis yielded a total of 1,090,735 effective reads from the fecal samples. The rarefaction and Shannon index curves tended to plateau, which indicated that the sequencing depth was reasonable and sufficient (Figures 4A,B). In addition, the Sobs index and Shannon index were significantly increased by GE treatment, particularly the low-dose treatment (Supplementary Figures S1A–B), which suggested that GE increased the richness and diversity of the gut microbiota. In addition, unweighted UniFrac-based PCoA of the beta diversity revealed a markedly clustered separation of the ND- and HFD-fed mice in based on the bacterial composition (Figure 4C). After GE intervention, the GEH samples were distinct from the ND and HFD samples. These results suggested that the intake of GE changed the overall microbial structure.

The relative abundances of predominant taxa were analyzed at the phylum level to clarify their changes. Bacteroides, Firmicutes, and Proteobacteria comprised the major populations of the gut microbiota in the fecal samples (Figure 4D). GE significantly increased the Bacteroidetes abundance and decreased the ratio of Firmicutes to Bacteroidetes (F/B) (Figures 4E,F). Moreover, a LEfSe analysis was performed to identify the alterations in the bacterial levels induced by HFD and GE intervention. As a result, 45 different OTUs from the three groups were listed together with the LDA score column and heatmap (Figures 4G,H). These OTUs were further analyzed using the Kruskal-Wallis H test. This study showed that 18 OTUs were significantly altered after HFD feeding and GE intervention. Compared with the ND group, the HFD mice displayed a significant decrease in the relative abundances of nine OTUs, and the changes in four of these OTUs were reversed by GEH intervention. In the GEH group, four OTUs were particularly enriched, and two OTUs were decreased. Notably, most of these changed OTUs belonged to Muribaculaceae, Faecalibaculum, Akkermansia, *Helicobacter*, Parabacteroides, Lachnospiraceae_NK4A136_group, and Ruminococcus_torques_group. Taken together, these data revealed that GE presented an appreciable capability to alter the gut microbiota profiles of HFD-fed mice.

### GE Intake Improves Gut Leak and Metabolic Endotoxemia in HFD-Fed Mice

**FIGURE 5:**
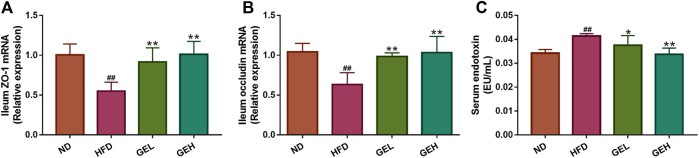
Effects of GE treatment on HFD-induced dyslipidemia, intestinal integrity and metabolic endotoxemia in HFD-fed mice. **(A–B)** Relative mRNA expression of ZO-1 and occludin in ileum tissues. **(C)** Circulating LPS levels. n = 8 per group. ^*#*^ *p* <0.05 or ^*##*^ *p* <0.01 vs. the ND group. *p <0.05 or **p <0.01 vs. the HFD group.

The HFD-induced changes in the gut microbiota enhance the intestinal permeability and the leakage of bacterial LPS into the circulation (Cani et al., 2008). Therefore, the expression of the genes encoding the tight junction proteins ZO-1 and occludin in the ileum and the LPS levels in the serum were monitored. Compared with the HFD group, the mice administered GE presented significantly increased mRNA expression levels of ZO-1 and occludin in ileum tissues, lowered circulating LPS levels and alleviated metabolic endotoxemia (Figure 5).

### Network Pharmacology Analysis Based on Serum Pharmacochemistry

**FIGURE 6:**
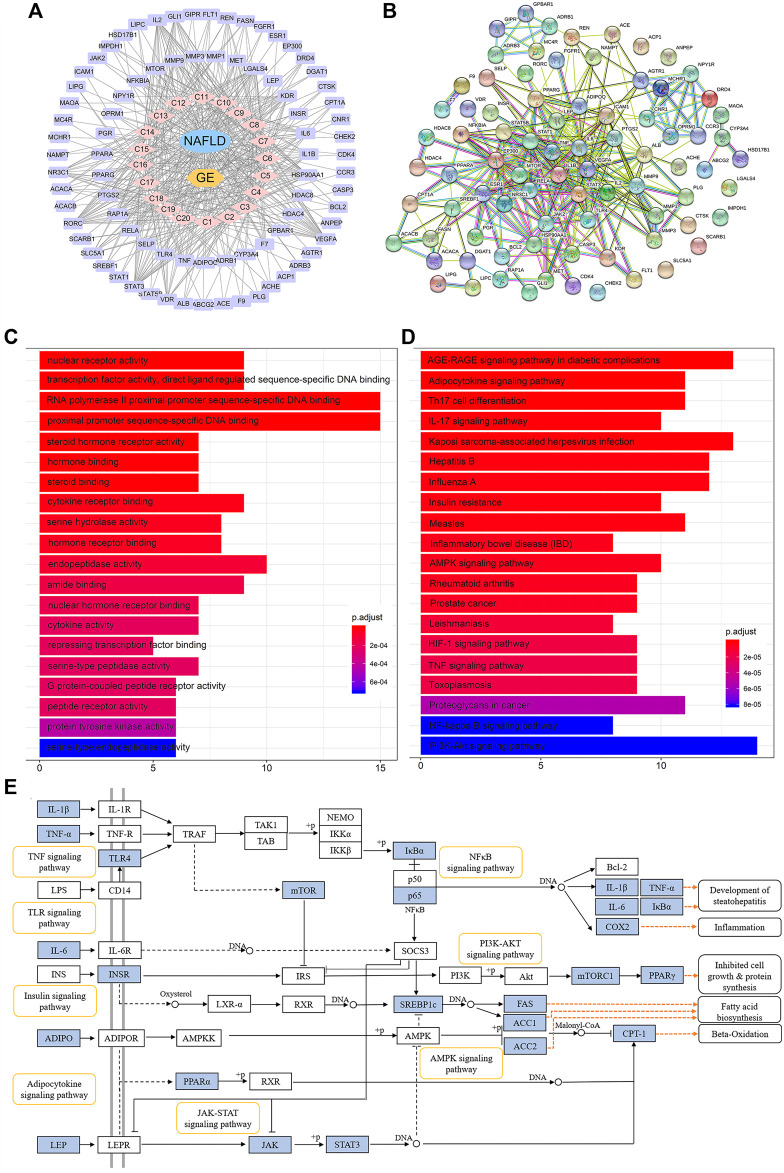
Network pharmacology analysis of the treatment of NAFLD with GE. **(A)** Disease-drug-component-target network. The lavender and pink circles represent targets and absorbed ingredients, respectively. A link indicates the interaction between nodes. **(B)** Protein-protein interaction network based on the STRING databases. The nodes represent proteins, and the edges represent protein-protein relationships. **(C)** GO terms of putative targets. **(D)** KEGG pathway terms of putative targets. In the GO and KEGG bar plots, the top 20 terms with *p*-value correction <0.05 are shown. **(E)** Representative signaling pathways mapping to putative targets. The blue nodes represent the putative targets of GE.

Because the absorption of a drug into the blood is one of the preconditions for its efficacy, UPLC-MS^n^ technology was used to detect the components in the blood after GE administration. A total of 20 components were identified, and these included ginsenoside Rg1 (C1), Re (C2), Rf (C3), Ra1 (C4), Ra3 (C5), Rb1 (C6), 20 (*S*)-Rg2 (C7), 20 (*S*)-Rh1 (C8), 20 (*R*)-Rg2 (C9), notoginsenoside Fc (C10), Rc (C11), 20 (*R*)-Rh1 (C12), Ra2 (C13), Rb2 (C14), Rb3 (C15), F1 (C16), Rd (C17), F2 (C18), 20 (*S*)-Rg3 (C19), and 20 (*R*)-Rg3 (C20) (Supplementary Figure S2). By mapping the targets of these components to disease targets, 82 putative targets were screened out (Supplementary Figure S3A). A disease-drug-component-target network was then established, and this network included 104 nodes and 986 interactions (Figure 6A). The median degree value of the absorbed component was 21, which suggested the synergistic actions of multiple components with multiple targets in GE. A topological data analysis revealed that 20 (*R*)-Rg2 exhibited the most interactions (degree = 26), followed by 20 (*R*)-Rh1 (degree = 25), 20 (*S*)-Rg3 (degree = 24), F2 and Rf (degree = 23), and F1, 20 (*S*)-Rh1, 20 (*S*)-Rg2 and Ra3 (degree = 22). The degrees of these components were greater than the median value; thus, the corresponding components were identified as the major effective constituents. Additionally, the PPI network reflected the interactions between the putative targets, which are represented by nodes (Figure 6B). According to previous reports, if the node degree is more than two-fold higher than the median degree of all nodes in the network, the node is considered a hub node (Xu et al., 2014). Thus, 22 hub nodes, including IL-6, TNF-α, IL-1β, leptin, adiponectin, signal transducer and activator of transcription 1 (STAT-1), STAT-3, RELA, TLR-4, and sterol-regulatory element binding protein 1c (SREBP-1c/SREBF1), were identified (Supplementary Figure S3B).

The GO functional analysis revealed that the highly enriched terms included nuclear receptor activity, gene and steroid-binding functions, cytokine activity, and transcription factor activity. The top 20 significant terms are shown in Figure 6C. The pathway enrichment analysis showed that 82 putative targets were frequently involved in energy metabolism and inflammation-related pathways, such as the adipocytokine signaling pathway, AMPK signaling pathway, insulin resistance, TNF signaling pathway, NF-κB signaling pathway, and phosphatidylinositol 3-kinase/protein kinase B (PI3K/Akt) pathway (Figure 6D). In addition, the inflammatory bowel disease (IBD) pathway was also enriched, which suggested that ginsenosides might be able to regulate the disturbance of the intestinal microbiota to a certain extent. Representative signaling pathways mapping to putative targets were integrated and presented in Figure 6E.

Remarkably, we demonstrated that GE reduced the serum LPS levels and ameliorated the lipid profile of HFD-fed mice. LPS triggers the inflammatory response (Cani et al., 2007), and AMPK plays a key role in regulating energy metabolism and the inflammatory state (O'Neill and Hardie, 2013). Therefore, we speculated that anti-inflammatory effects and regulation of the metabolic balance might be responsible for the effects of GE on NAFLD and were further investigated.

### GE Intake Improves Inflammation in HFD-Fed Mice

**FIGURE 7:**
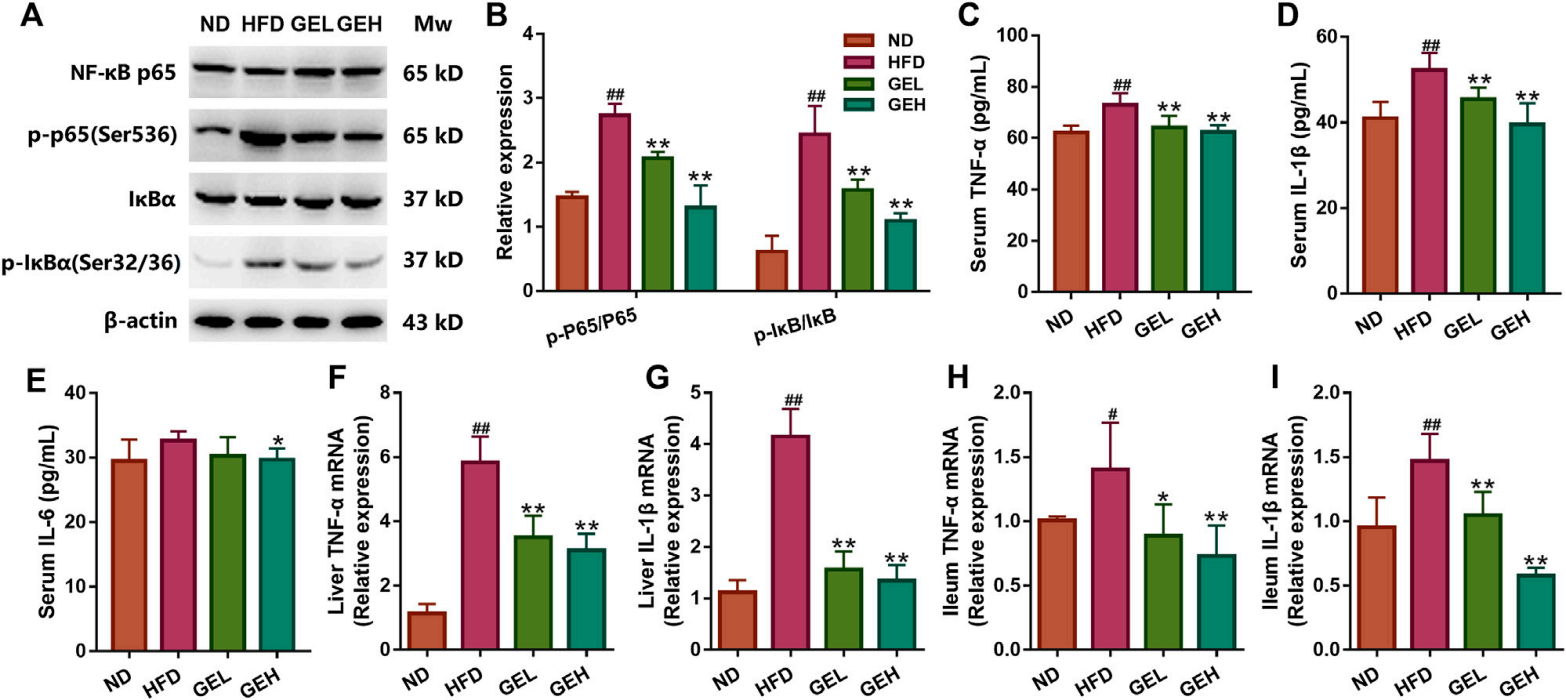
Effects of GE treatment on inflammation in HFD-fed mice. **(A–B)** Phosphorylation levels of NF-κB p65 (Ser 536) and IκB (Ser 32/36) in total liver homogenates. The bands were quantified, and the values are relative to the *β*-actin levels (loading control). **(C–E)** Circulating TNF-α, IL-1β and IL-6 levels. **(F–I)** Relative mRNA expression of TNF-α and IL-1β in ileum and liver tissues. n = 8 per group. ^*#*^ *p* <0.05 or ^*##*^ *p* <0.01 vs. the ND group. *p <0.05 or **p <0.01 vs. the HFD group.

Because LPS can activate the TLR4/NF-κB signaling pathway (Soares et al., 2010), we assessed whether NF-κB p65 and IκB activation were affected by GE intake (Figures 7A,B and Supplementary Figure S4). Western blotting analysis showed that the phosphorylation levels of IκB and NF-κB p65 were significantly higher in the HFD group, but these changes were reversed after GE treatment. Because enhanced activation of NF-κB/IκB pathways induces the production of proinflammatory cytokines, including TNF-α, IL-1β and IL-6 (Hayden and Ghosh, 2008), we examined the above-mentioned cytokines in serum and target tissues (Figures 7C–I). The serum levels of TNF-α and IL-1β, and the mRNA levels of TNF-α and IL-1β in hepatic and ileum tissues were obviously higher in HFD-fed mice than in ND mice. The levels of these cytokines were down-regulated in a dose-dependent manner by GE supplementation. Although there was a slight rise in serum IL-6 levels in the HFD group compared with the ND group, supplementation with 200 mg/kg GE significantly decreased serum IL-6 levels. These results indicate that GE reduces systemic and local inflammation by inhibiting the NF-κB/IκB signaling pathway.

### GE Intake Regulates Lipid Metabolism-Related Genes and Serum Hormones

**FIGURE 8:**
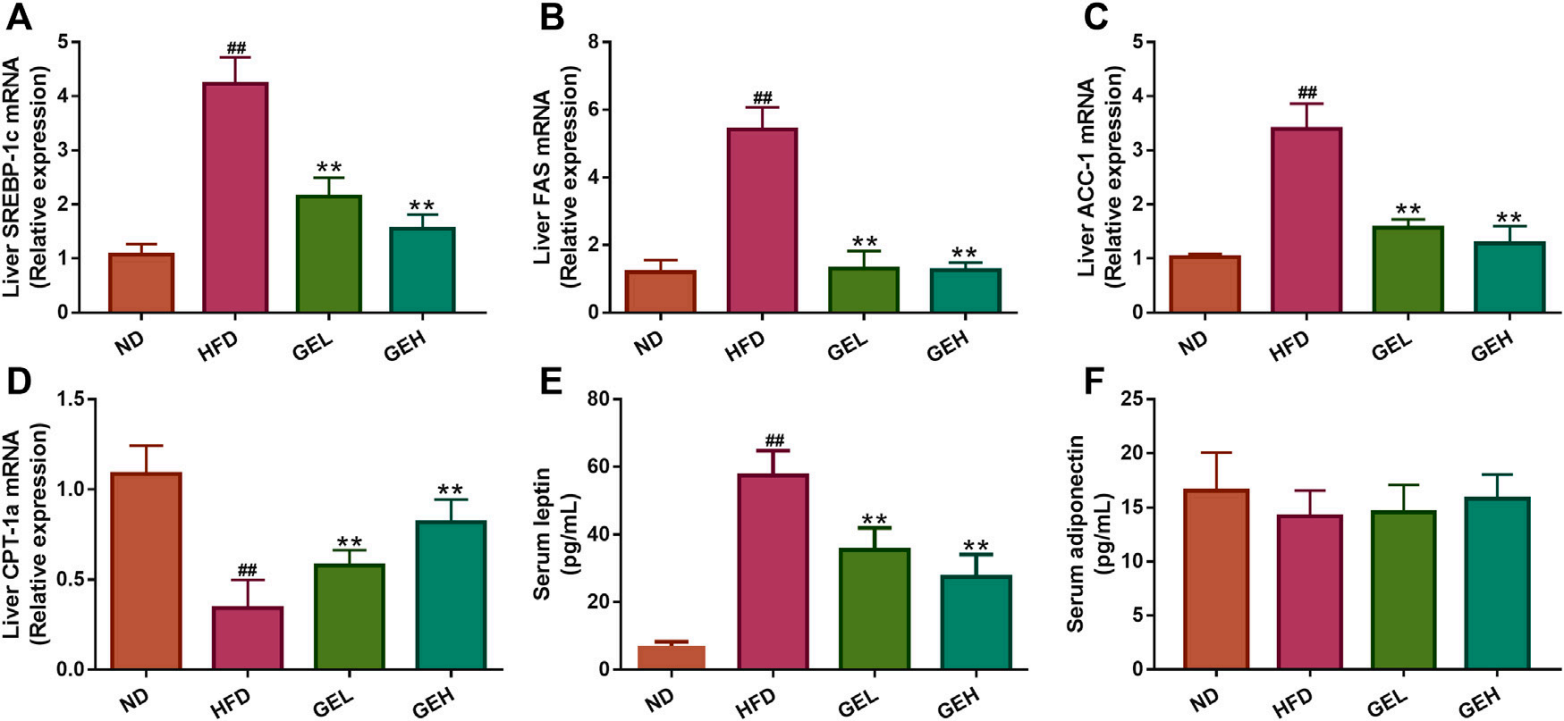
Effects of GE treatment on hepatic gene expression and serum hormones involved in lipid metabolism. **(A–C)** The mRNA relative expression of adipolytic genes in the liver. **(D)** The mRNA relative expression of adipogenic gene in the liver. **(E–F)** The levels of serum leptin and adiponectin. n = 8 per group. ^*#*^ *p* <0.05 or ^*##*^ *p* <0.01 vs. the ND group. *p <0.05 or **p <0.01 vs. the HFD group.

The predicted putative targets related to fatty acid oxidation and synthesis obtained from the network pharmacology analysis, including SREBP-1c, fatty acid synthase (FAS), acetyl-CoA carboxylase 1 (ACC-1), and carnitine palmitoyltransferase 1a (CPT-1a), were validated. As shown in Figures 8A–D, GE supplementation significantly reduced the mRNA expression levels of SREBP-1c, FAS and ACC-1 but increased the CPT-1a expression level. The above-described results suggested that GE mitigated hepatic lipid accumulation by promoting adipolytic gene expression and restraining adipogenic gene expression. Leptin and adiponectin are adipocyte-derived polypeptide hormones involved in the regulation of energy homeostasis (Oswal and Yeo, 2010; Fang and Judd, 2018). Under long-term GE treatment, the GEL and GEH mice showed apparent alterations in their abnormal serum hormone leptin levels but exhibited no change in their adiponectin content (Figures 8E,F).

### Correlation Between NAFLD Traits and Key Gut Microbiota

**FIGURE 9:**
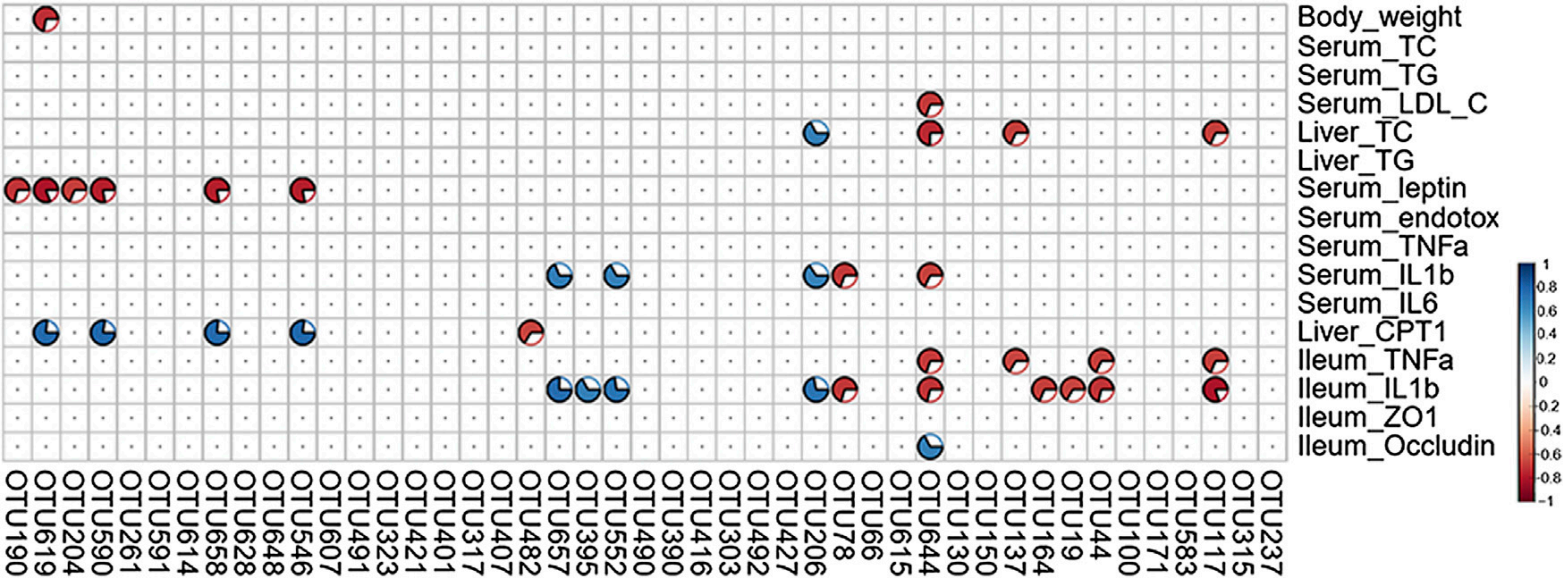
Heatmap of Spearman’s correlation analysis between key intestinal microbiota and NAFLD traits. The intensity of the color represents the degree of association (red, negative correlation; blue, positive correlation). Significant correlations are annotated by *p* <0.05 and absolute value of the correlation coefficient >0.5.

A total of 16 NAFLD-related parameters were screened by the VIF analysis. To explore the relationships between key bacteria and the above-mentioned disease parameters, a Spearman’s correlation analysis was performed to calculate the correlation coefficients. The generated heatmap showed that several NAFLD traits were significantly correlated with the levels of different OTUs (Figure 9). OTU190/619/204/658 (Muribaculaceae) displayed negative correlations with the serum leptin levels, and OTU619/658 also exhibited positive correlations with the hepatic CPT-1a levels. Additionally, OTU137 (Muribaculaceae) was negatively correlated with the hepatic TC and ileum TNF-α levels. OTU395 (Lachnospiraceae_NK4A136_group) was positively correlated with the ileum IL-1β levels, and OTU482 (Lachnospiraceae) was negatively correlated with the liver CPT-1a levels. OTU644 (Parabacteroides) was negatively correlated with most inflammation-associated traits (such as the serum IL-1β, ileum TNF-α and ileum IL-1β levels) and positively correlated with the levels of occludin, which protects the function of the intestinal barrier. These results suggest that the above-mentioned members of the gut microbiota mediate the benefits of GE on NAFLD.

## Discussion

The preventive effects of ginsenosides on HFD-induced NAFLD have been investigated in many studies (Park et al., 2020; Xu et al., 2020), but the underlying mechanisms remain to be elucidated due to the multicomponent and multitarget features. In this study, GE administration ameliorated HFD-induced NAFLD by modulating the gut microbiota, improving the intestinal integrity, regulating energy homeostasis, and alleviating inflammation, and these effects were related to its rational integration of ginsenosides.

The gut microbiota is considered a potential target for NAFLD treatment (Kolodziejczyk et al., 2019). We found that GE enhanced the diversity of the bacterial community and produced a dramatic shift in the gut microbial community composition by decreasing the F/B ratio. However, an increased F/B ratio helps to promote obesity and related metabolic diseases, and increase energy harvest and low-grade inflammation (Ley et al., 2005; Clemente et al., 2012). The results suggested that the effect of GE on relieving NAFLD was associated with regulating the F/B ratio. Previous studies have shown that Parabacteroides alleviates NAFLD and dysfunctions *via* the production of succinate and secondary bile acids and the activation of FXR pathways in the gut (Wang et al., 2019). Muribaculaceae is a probiotic bacterium that produces short-chain fatty acids (SCFAs) and metabolizes mucin *O*-glycan to maintain the dynamic balance of the intestinal mucus barrier and assist microbial colonization (Ndeh and Gilbert, 2018; Lee et al., 2019). Interestingly, OTU644 (belonging to Parabacteroides) and OTU619/190/137 (belonging to Muribaculaceae) were markedly decreased by HFD feeding, and these trends were reversed by GE administration. Besides the above bacteria are negatively correlated with many host NAFLD parameters, including the serum leptin levels, body weight, hepatic TG levels and ileum TNF-α levels, which suggests that GE exerts a probiotic effect. Notably, we also found that GE induced a significant increase in the proliferation of Akkermansia (OTU237) and Ruminococcus_torques_group (OTU66), which are generally considered beneficial microbiota for modulating metabolic homeostasis (Everard et al., 2013; Zhang et al., 2019). Bacteria within the family Lachnospiraceae promote the susceptibility of obesity and intestinal LPS into the blood (Kameyama and Itoh, 2014). Based on the present study, GE-inhibited blooms of OTU395/421 (Lachnospiraceae_NK4A136_group) and OTU482 (Lachnospiraceae) might reduce LPS production in HFD-fed mice. GE intervention also significantly reversed the HFD-induced increases in species of *Helicobacter* (OTU303), a type of infectious and pathogenic bacteria that shows increased relative abundances in HFD-fed mice (Jiao et al., 2019; Ma et al., 2019). Furthermore, the correlation analysis showed that TNF-α and IL-1β exhibited significantly negative correlations with Parabacteroides and Muribaculaceae and positive correlations with Lachnospiraceae. This result suggested that the regulatory effect of GE on these bacteria was helpful for improving the inflammatory status, which was consistent with the results of the network pharmacology analysis and validation experiments, and reflected the synergistic and multiple mechanisms related to GE intervention. Taken together, these results demonstrated that the effect of GE on dietary-induced NAFLD is achieved by modulating the gut microbiota, particularly by facilitating the prevalence of beneficial bacteria (Parabacteroides, Muribaculaceae, Akkermansia, and Ruminococcus_torques_group) and decreasing the prevalence of harmful bacteria (Lachnospiraceae and *Helicobacter*).

The altered microbial community in obesity and NAFLD increases intestinal permeability and promotes the leakage of bacterial LPS into the systemic circulation (Cani et al., 2008). In this study, GE significantly prevented metabolic endotoxemia and restored intestinal integrity by boosting the mRNA expression of two tight-junction proteins (ZO-1 and occludin) in HFD-fed mice, which indicated that the ability of GE to relieve NAFLD was attributed to the blunting of LPS leakage and repair of the intestinal barrier. In addition, these results suggested the subsequent inhibition of LPS-mediated systemic inflammation.

It is well known that active ingredients dwelling in the intestinal tract are absorbed into the blood through intestinal epithelial cells and then selectively interact with multiple targets to treat diseases (Zhao et al., 2015; Xu et al., 2017). As observed in the current study, 20 components were absorbed into the bloodstream after GE administration. A total of 82 putative targets of these components were identified by network pharmacology analysis, and the enriched pathways were mainly associated with inflammation and energy metabolism, including TNF-α signaling, NF-κB signaling, AMPK signaling and adipocytokine signaling pathway. Interestingly, a dynamic crosstalk between inflammation and energy homeostasis are important for the pathophysiology of NAFLD and its progression (Friedman et al., 2018; Polyzos et al., 2019).

On the one hand, an increase of bacteria-derived LPS in the systemic circulation initiates systemic inflammatory responses by activating TLR4/NF-κB signaling and then releasing proinflammatory cytokines (Cani et al., 2007; Winer et al., 2016). These factors could mediate insulin resistance, hepatic steatosis and even fibrosis in NAFLD (Tomita et al., 2006; Buzzetti et al., 2016). Given that metabolic endotoxemia was reduced after GE intervention, we selected the key targets predicted on the NF-κB signaling pathway for experimental verification. Remarkably, the phosphorylations of IκB-α and NF-κB p65 proteins were suppressed in hepatic tissues, and the productions of TNF-α, IL-1β and IL-6 were decreased in the circulation and liver after GE treatment, indicating the beneficial anti-NAFLD effect of GE was at least partially attributed to the inhibition of systemic and local inflammation.

On the other hand, lipid metabolic disorder causes hepatocellular inflammation and downstream cytokine production (Cai et al., 2005; Hotamisligil, 2006). Although AMPK, an important sensor in the control of lipid homeostasis, was not fished by network pharmacology analysis, the upstream targets (leptin and adiponectin) and downstream targets (SREBP-1c, FAS, ACC-1, and CPT1a) for AMPK were all predicted for the anti-NAFLD effect of GE. It is well known that adipocytokine leptin inhibits appetite and weight gain, yet leptin resistance (hyperleptinemia) is commonly observed in NAFLD individuals (Oswal and Yeo, 2010; Polyzos et al., 2015). GE significantly the reduced serum leptin levels and restored leptin sensitivity, which were partly responsible for a small reduction in energy intake and weight loss observed in GE-fed mice. Besides adiponectin has been demonstrated to stimulate fatty acid oxidation and glucose utilization in liver and muscle (Fang and Judd, 2018), but its levels had no significant changes after GE treatment. SREBP-1c, a dominant transcription factor, regulates the fatty acid synthesis genes such as FAS and ACC-1 (O'Neill and Hardie, 2013). Moreover, CPT-1 is a rate-limiting enzyme for the transfer of long-chain fatty acids into mitochondria for fatty acid *β*-oxidation (Kahn et al., 2005). In this study, the mRNA expression levels both of SREBP-1c, FAS and ACC-1 were significantly downregulated, while the mRNA levels of CPT-1a were obviously upregulated by GE, which contributed to regulate lipid homeostasis in NAFLD. In particular, excess fatty acid delivered to the liver from blood following dysregulated lipolysis in adipose tissue could lead to the activation of c-Jun N-terminal kinase-activator protein-1 and inhibitor of NF-κB kinase-NF-κB inflammatory pathways, which are critically involved in the development of the chronic inflammatory state in NAFLD (Cai et al., 2005; Hotamisligil, 2006; Buzzetti et al., 2016). GE ameliorated ectopic fat accumulation in the liver by regulating lipolytic and lipogenic gene expression as we described previously, suggesting the fatty acid-mediated activation of NF-κB would be inhibited by GE. Additionally, leptin increases proinflammatory cytokine production by acting on the Janus kinase 2/STAT-3 and PI3K/Akt/mammalian target of rapamycin pathways (Monteiro et al., 2019). In the present study, the levels of proinflammatory cytokines were decreased in GE-fed mice, and the above two pathways were computationally predicted to account for the action of GE by network pharmacology. Thus, the regulation of leptin by GE was also a contributor to the beneficial effects against NAFLD by suppressing inflammation. Further in-depth studies are warranted to investigate the underlying mechanisms based on the current results.

What’s more, gut microbiota-derived metabolites are key factors in host-microbiota cross-talk, such as SCFAs, bile acids, branched-chain amino acids, trimethylamine N-oxide, imidazole propionate, tryptophan and indole derivatives (Agus et al., 2020). Intriguingly, the intestinal bacteria modulated by GE were mainly related to the SCFAs production in our study, including Muribaculaceae and Akkermansia. Among them, Muribaculaceae, also known as “S24–7”, specially produces acetate and propionate (Smith et al., 2019), and Akkermansia could produce acetate, propionate and butyrate (Belzer and de Vos, 2012). They were promoted the growth after GE administration, suggesting the generation of acetate, propionate and butyrate might be increased in GE-fed mice. Studies have demonstrated that butyrate provides energy for colonic epithelial cells, ameliorates lipid accumulation and liver inflammation to resist NAFLD (Kolodziejczyk et al., 2019). Acetate binds G-protein-coupled receptor 43 and propionate primarily recognizes G-protein-coupled receptor 41, which inhibit appetite and stimulate insulin secretion to regulate energy metabolism (Boulangé et al., 2016; Kolodziejczyk et al., 2019). Therefore, we speculated that the changes in SCFAs mediated by the gut microbiota might play a positive role in the benefits of GE of energy metabolism and inflammation in NAFLD. Subsequently, we would further apply metabolomics to explore the interactions between intestinal bacteria metabolites, especially SCFAs, and the host in the anti-NAFLD effect of GE.

## Conclusion

In summary, the present study demonstrated that GE ameliorated HFD-induced NAFLD by maintaining the energy balance, modulating gut dysbiosis, and improving the intestinal integrity and metabolic inflammation. Specifically, the GE-mediated synergistic modulation of LPS- and SCFA-producing bacteria, dysbiosis-mediated metabolic endotoxemia and LPS-mediated NF-κB/IκB signaling played a vital role in the effect of GE on NAFLD. Additionally, the above-described health benefits were due to the rational integration of ginsenosides. In this study, a strategy combining bacterial gene sequencing, serum pharmacochemistry and network pharmacology was used to explore the action of multicomponent natural extracts, and the results suggested that GE can serve as a functional agent to balance the gut microbial ecosystem and prevent NAFLD and metabolic inflammation.

## Data Availability

The datasets presented in this study can be found in online repositories. The names of the repository/repositories and accession number(s) can be found below: NCBI SRA database, https://www.ncbi.nlm.nih.gov/sra, PRJNA673766.
