# Supplementary material for: Ginsenosides Improve Nonalcoholic Fatty Liver Disease via Integrated Regulation of Gut Microbiota, Inflammation and Energy Homeostasis
Source: Front Pharmacol. 2021 Feb 12;12:622841. doi: 10.3389/fphar.2021.622841 (PMC7928318; doi:10.3389/fphar.2021.622841)
Supplement: Supplementary file 1 [file datasheet1.docx]

Supplementary Information

# 1 Preparation of Ginsenoside Extract (GE)

The dried roots and rhizomes of *Panax ginseng* C. A. Mey. were purchased from Fushun, Jilin, China. These materials were authenticated by Prof. Xiangyan Li (Changchun University of Chinese Medicine, Changchun, Jilin, China) , and voucher specimens (No. B20171120) was stored in their Research Laboratory.

GE was prepared and provided by Changchun University of Chinese Medicine, Changchun, Jilin, China. The detailed preparation process was as follows. The dried roots and rhizomes of *Panax ginseng* C. A. Mey. (5 kg) were refluxed with water (1:10, *w/v*) for 2 h. The filtrates were collected and the residues were then refluxed in water (1:10, *w/v*) for 1.5 h. Two batches of filtrates were combined and concentrated using a rotary evaporator. Next, the concentrated solution was added on a loading-treated D101 macroreticular adsorption resin column and washed with distilled water and 60% ethanol, respectively. The collected 60% ethanol-eluted part was evaporated and dried under vacuum freezer to obtain GE.

# 2 Chemical Characterization of GE

## 2.1 Qualitative Analysis of GE

The GE sample was dissolved in 70% methanol and the concentration was 2 mg/mL. The solution was filtered through a 0.22-µm microporous filter before injection for UPLC-MS^n^ analysis. Electron spray ionization (ESI) hybrid linear ion trap quadrupole-Orbitrap mass spectrometer was used for phytochemical analysis of GE, coupled with a Thermo Accela 600 HPLC system (Thermo Scientific, Bremen, Germany). The ingredients were separated on an ACQUITY HSS T3 C18 UPLC column (100 mm × 2.1 mm i.d., 1.8 µm) at 30 °C. Acetonitrile (A) and 0.1% (*v/v*) formic acid in water (B) were as elution solvents at a flow rate of 0.3 mL/min and the gradient program was as follows: 0-1 min, 20% A; 1-10 min, 20-35% A; 10-22 min, 35-70% A; 22-24 min, 70-90% A; 24-26 min, 90% A; 26-27 min, 90-20% A; 27-30 min, 20% A. The injection volume was 2 µL. Mass spectra was acquired in negative ionization mode with a scan range of *m/z* 200-1500. The instrument was operated under the following setting parameters: capillary voltage, -35 V; source voltage, 3 kV; tube lens voltage, -110 V; capillary temperature, 350 °C; sheath gas (nitrogen) flow, 40 arb. and auxiliary gas (nitrogen) flow, 20 arb. Xcalibur 3.0 software was used for tentative peak identification.

As shown in Figure 1-2 and Table 1, a total of 56 components in GE were identified according to available standards, retention time, accurate *m/z* values within 5 ppm error, abundant fragment ions and related literature reports (Li et al., 2010; Xie et al., 2012; Qiu et al., 2015).


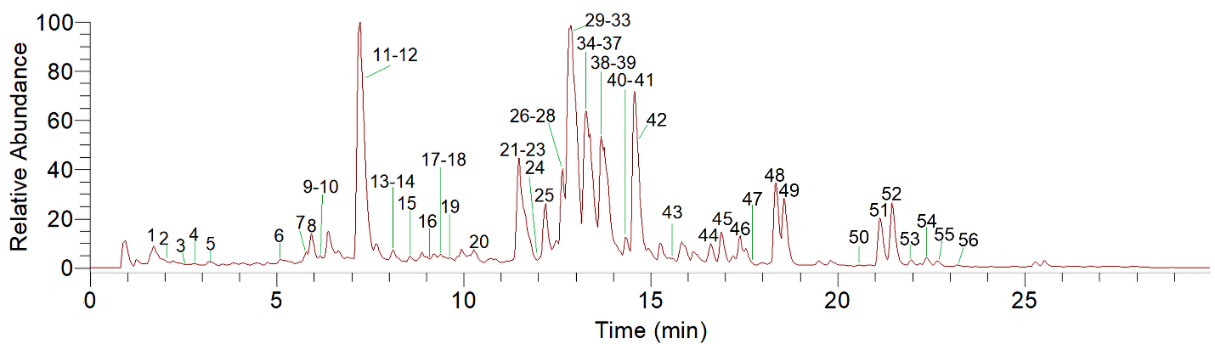


**Figure 1.** Total ion chromatogram of GE in negative ion mode. The peak number corresponds to the compound number in Table 1.

**
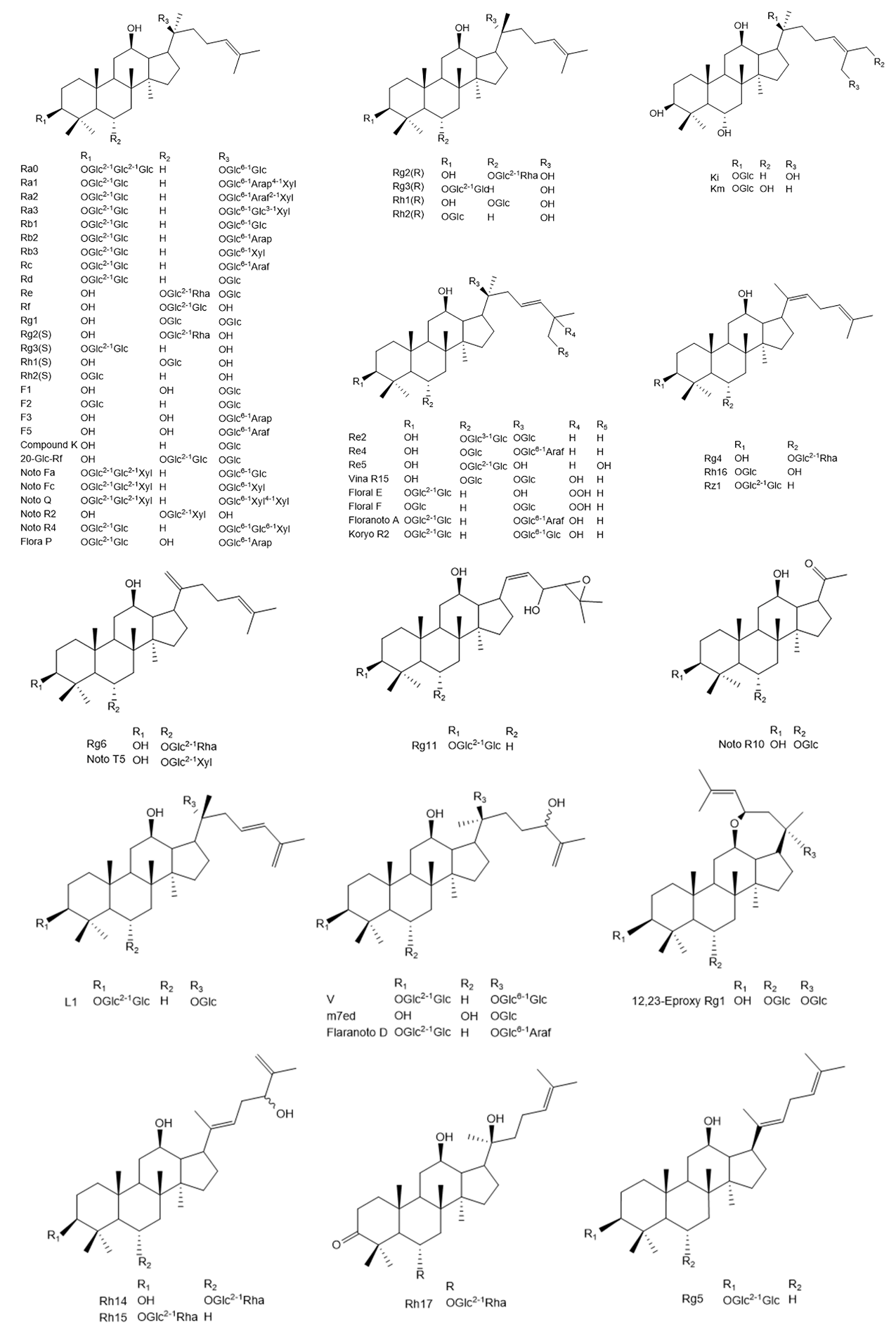
**

**Figure 2.** Structures of components identified in GE.

**Table 1** Components Identified of GE by UPLC LTQ-Orbitrap analysis.

| No. | *t*_R_  (min) | Identification | Molecular  formula | Proposal ions | Theoretical  mass (*m/z*) | Experimental  mass (*m/z*) | Mass error (ppm) | MS^2^ fragment ions (*m/z*) |
| --- | --- | --- | --- | --- | --- | --- | --- | --- |
| 1 | 1.67 | Vinaginsenoside R15 | C_42_H_72_O_15_ | [M-H+HCOOH]^-^ | 861.4842 | 861.4866 | 2.791 | 815.3403 [M-H]^-^, 653.4201 [M-H-Glc]^-^, 491.2797 [M-H-2Glc]^-^ |
| 2 | 2.25 | Ginsenoside Re5 | C_42_H_72_O_15_ | [M-H+HCOOH]^-^ | 861.4842 | 861.4866 | 2.720 | 815.4063 [M-H]^-^, 653.3999 [M-H-Glc]^-^, 491.3277 [M-H-2Glc]^-^ |
| 3 | 2.54 | Floralginsenoside F | C_42_H_72_O_15_ | [M-H+HCOOH]^-^ | 861.4842 | 861.4857 | 1.733 | 815.5444 [M-H]^-^, 653.4222 [M-H-Glc]^-^, 491.7540 [M-H-2Glc]^-^ |
| 4 | 2.72 | Notoginsenoside R10 | C_30_H_50_O_9_ | [M-H+HCOOH]^-^ | 599.3426 | 599.3445 | 3.156 | 553.3175 [M-H]^-^, 391.2295 [M-H-Glc]^-^ |
| 5 | 3.37 | Floralginsenoside E | C_42_H_72_O_15_ | [M-H+HCOOH]^-^ | 861.4842 | 861.4851 | 1.028 | 815.3965 [M-H]^-^, 653.4998 [M-H-Glc]^-^, 491.4325 [M-H-2Glc]^-^ |
| 6 | 5.02 | Floralginsenoside P | C_53_H_90_O_23_ | [M-H]^-^ | 1093.5789 | 1093.5806 | 1.513 | 961.5354 [M-H-Xyl]^-^, 799.6251 [M-H-Xyl-Glc]^-^, 637.5382 [M-H-Xyl-2Glc]^-^, 475.5466 [M-H-Xyl-3Glc]^-^ |
| 7 | 5.69 | Ginsenoside Km | C_36_H_62_O_10_ | [M-H+HCOOH]^-^ | 699.4314 | 699.4329 | 2.186 | 653.4079 [M-H]^-^, 491.3725 [M-H-Glc]^-^ |
| 8 | 5.92 | 20-*O*-glucosylginsenoside Rf | C_48_H_82_O_19_ | [M-H+HCOOH]^-^ | 1007.5421 | 1007.5447 | 2.525 | 799.4959 [M-H-Glc]^-^, 637.4606 [M-H-2Glc]^-^, 475.4870 [M-H-3Glc]^-^ |
| 9 | 6.13 | Ginsenoside Ki | C_36_H_62_O_10_ | [M-H+HCOOH]^-^ | 699.4314 | 699.4331 | 2.440 | 653.4291 [M-H]^-^, 491.3811 [M-H-Glc]^-^ |
| 10 | 6.19 | Ginsenoside Re4 | C_47_H_80_O_18_ | [M-H+HCOOH]^-^ | 977.5316 | 977.5344 | 2.914 | 799.4558 [M-H-Araf]^-^, 637.4622 [M-H-Araf-Glc]^-^, 475.5133 [M-H-Araf-2Glc]^-^ |
| 11^a^ | 7.16 | Ginsenoside Rg1 | C_42_H_72_O_14_ | [M-H+HCOOH]^-^ | 845.4893 | 845.4914 | 2.457 | 799.3745 [M-H]^-^, 637.3869 [M-H-Glc]^-^, 475.4484 [M-H-2Glc]^-^ |
| 12^a^ | 7.22 | Ginsenoside Re | C_48_H_82_O_18_ | [M-H+HCOOH]^-^ | 991.5472 | 991.5490 | 1.804 | 799.5438 [M-H-Rha]^-^, 637.4913 [M-H-Rha-Glc]^-^, 475.5300 [M-H-Rha-2Glc]^-^ |
| 13 | 8.16 | Ginsenoside M7ed | C_36_H_62_O_10_ | [M-H+HCOOH]^-^ | 699.4314 | 699.4336 | 3.126 | 653.3935 [M-H]^-^, 491.4491 [M-H-Glc]^-^ |
| 14 | 8.25 | 12,23-Eproxyginsenoside Rg1/ginsenoside Rg11 | C_42_H_70_O_14_ | [M-H+HCOOH]^-^ | 843.4737 | 843.4760 | 2.712 | 797.3747 [M-H]^-^, 635.3777 [M-H-Glc]^-^, 473.4807 [M-H-2Glc]^-^ |
| 15 | 8.56 | Koryoginsenoside R2 | C_54_H_92_O_24_ | [M-H+HCOOH]^-^ | 1169.5950 | 1169.5972 | 1.890 | 961.6059 [M-H-Glc]^-^, 799.5601 [M-H-2Glc]^-^, 781.4884 [M-H-2Glc-H_2_O]^-^,  619.6362 [M-H-3Glc-H_2_O]^-^,  475.4835 [M-H-4Glc]^-^ |
| 16 | 9.05 | Ginsenoside V | C_54_H_92_O_24_ | [M-H+HCOOH]^-^ | 1169.5950 | 1169.5974 | 2.096 | 961.4642 [M-H-Glc]^-^, 799.5772 [M-H-2Glc]^-^, 781.5293 [M-H-2Glc-H_2_O]^-^,  637.4578 [M-H-3Glc]^-^, 475.4693 [M-H-4Glc]^-^ |

**Table 1** (*Continued*)

| No. | *t*_R_  (min) | Identification | Molecular  formula | Proposal ions | Theoretical  mass (*m/z*) | Experimental  mass (*m/z*) | Mass error (ppm) | MS^2^ fragment ions (*m/z*) |
| --- | --- | --- | --- | --- | --- | --- | --- | --- |
| 17 | 9.36 | Floranotoginsenoside A/D | C_53_H_90_O_23_ | [M-H+HCOOH]^-^ | 1139.5844 | 1139.5874 | 2.638 | 961.5539 [M-H-Araf]^-^, 799.4678 [M-H-Araf-Glc]^-^, 781.5451 [M-H-Araf-Glc-H_2_O]^-^, 637.4504 [M-H-Araf-2Glc]^-^, 475.5069 [M-H-Araf-3Glc]^-^ |
| 18 | 9.53 | Ginsenoside F3 | C_41_H_70_O_13_ | [M-H+HCOOH]^-^ | 815.4787 | 815.4813 | 3.081 | 769.4373 [M-H]^-^, 637.5117 [M-H-Arap]^-^, 475.5050 [M-H-Arap-Glc]^-^ |
| 19 | 9.70 | Floranotoginsenoside A/D | C_53_H_90_O_23_ | [M-H+HCOOH]^-^ | 1139.5844 | 1139.5875 | 2.743 | 961.5292 [M-H-Araf]^-^, 799.4843 [M-H-Araf-Glc]^-^, 781.4879 [M-H-Araf-Glc-H_2_O]^-^, 637.4685 [M-H-Araf-2Glc]^-^, 475.3780 [M-H-Araf-3Glc]^-^ |
| 20 | 10.27 | Ginsenoside Re2 | C_48_H_82_O_19_ | [M-H+HCOOH]^-^ | 1007.5421 | 1007.5449 | 2.704 | 799.4870 [M-H-Glc]^-^, 637.5974 [M-H-2Glc]^-^, 475.5080 [M-H-3Glc]^-^ |
| 21^a^ | 11.48 | Ginsenoside Rf | C_42_H_72_O_14_ | [M-H+HCOOH]^-^ | 845.4893 | 845.4917 | 2.824 | 799.4360 [M-H]^-^, 637.4255 [M-H-Glc]^-^, 475.4641 [M-H-2Glc]^-^ |
| 22 | 11.66 | Notoginsenoside R4 | C_59_H_100_O_27_ | [M-H]^-^ | 1239.6368 | 1239.6370 | 0.110 | 1107.5050 [M-H-Xly]^-^, 945.5491 [M-H-Xly-Glc]^-^, 783.4801 [M-H-Xly-2Glc]^-^,  621.4962 [M-H-Xly-3Glc]^-^, 459.4197 [M-H-Xly-4Glc]^-^ |
| 23 | 11.77 | Notoginsenoside Q | C_63_H_106_O_30_ | [M-H]^-^ | 1341.6685 | 1341.6696 | 0.777 | 1209.6189 [M-H-Xyl]^-^, 1077.4733 [M-H-2Xyl]^-^, 945.6221 [M-H-3Xyl]^-^, 783.4782 [M-H-3Xyl-Glc]^-^, 621.6181 [M-H-3Xyl-2Glc]^-^, 459.5977 [M-H-3Xyl-3Glc]^-^ |
| 24 | 12.06 | Ginsenoside Ra0 | C_60_H_102_O_28_ | [M-H]^-^ | 1269.6474 | 1269.6497 | 1.789 | 1107.5471 [M-H-Glc]^-^, 945.5252 [M-H-2Glc]^-^, 783.4638 [M-H-3Glc]^-^, 621.5610 [M-H-4Glc]^-^, 459.3991 [M-H-5Glc]^-^ |
| 25 | 12.19 | Ginsenoside F5 | C_41_H_70_O_13_ | [M-H]^-^ | 769.4733 | 769.4736 | 0.470 | 637.4535 [M-H-Araf]^-^, 475.4343 [M-H-Araf-Glc]^-^ |
| 26 | 12.52 | Notoginsenoside Fa | C_59_H_100_O_27_ | [M-H]^-^ | 1239.6368 | 1239.6387 | 1.498 | 1107.5479 [M-H-Xly]^-^, 945.5511 [M-H-Xly-Glc]^-^, 783.5809 [M-H-Xly-2Glc]^-^, 621.5145 [M-H-Xly-3Glc]^-^, 459.3889 [M-H-Xly-4Glc]^-^ |
| 27 | 12.55 | Notoginsenoside R2 | C_41_H_70_O_13_ | [M-H+HCOOH]^-^ | 815.4787 | 815.4808 | 2.480 | 769.4207 [M-H]^-^, 637.5449 [M-H-Xyl]^-^, 475.5504 [M-H-Xyl-Glc]^-^ |

**Table 1** (*Continued*)

| No. | *t*_R_  (min) | Identification | Molecular  formula | Proposal ions | Theoretical  mass (*m/z*) | Experimental  mass (*m/z*) | Mass error (ppm) | MS^2^ fragment ions (*m/z*) |
| --- | --- | --- | --- | --- | --- | --- | --- | --- |
| 28 | 12.65 | Ginsenoside Ra1 | C_58_H_98_O_26_ | [M-H]^-^ | 1209.6263 | 1209.6259 | -0.338 | 1077.5234 [M-H-Xyl]^-^, 945.4890 [M-H-Xyl-Arap]^-^, 783.5171 [M-H-Xyl-Arap-Glc]^-^, 621.4638 [M-H-Xyl-Arap-2Glc]^-^, 459.3909 [M-H-Xyl-Arap-3Glc]^-^ |
| 29 | 12.75 | Ginsenoside Ra3 | C_59_H_100_O_27_ | [M-H]^-^ | 1239.6368 | 1239.6375 | 0.505 | 1107.5942 [M-H-Xly]^-^, 945.4899 [M-H-Xly-Glc]^-^, 783.5617 [M-H-Xly-2Glc]^-^, 621.5550 [M-H-Xly-3Glc]^-^, 459.5076 [M-H-Xly-4Glc]^-^ |
| 30^a^ | 12.80 | Ginsenoside Rb1 | C_54_H_92_O_23_ | [M-H]^-^ | 1107.5946 | 1107.5955 | 0.808 | 945.6096 [M-H-Glc]^-^, 783.6151 [M-H-2Glc]^-^, 621.4423 [M-H-3Glc]^-^, 459.5066 [M-H-4Glc]^-^ |
| 31^a^ | 12.82 | 20(*S*)-ginsenoside Rg2 | C_42_H_72_O_13_ | [M-H+HCOOH]^-^ | 829.4944 | 829.4981 | 4.475 | 637.4955 [M-H-Rha]^-^, 475.5246 [M-H-Rha-Glc]^-^ |
| 32^a^ | 12.99 | 20(*S*)-ginsenoside Rh1 | C_36_H_62_O_9_ | [M-H+HCOOH]^-^ | 683.4365 | 683.4389 | 3.528 | 637.4667 [M-H]^-^, 475.3743 [M-H-Glc]^-^ |
| 33 | 13.02 | 20(*R*)-ginsenoside Rg2 | C_42_H_72_O_13_ | [M-H+HCOOH]^-^ | 829.4944 | 829.4970 | 3.194 | 637.4955 [M-H-Rha]^-^, 475.5246 [M-H-Rha-Glc]^-^ |
| 34 | 13.22 | Notoginsenoside Fc | C_58_H_98_O_26_ | [M-H]^-^ | 1209.6263 | 1209.6273 | 0.877 | 1077.5496 [M-H-Xyl]^-^, 945.5019 [M-H-2Xyl]^-^, 783.4934 [M-H-2Xyl-Glc]^-^, 621.5859 [M-H-2Xyl-2Glc]^-^, 459.4782 [M-H-2Xyl-3Glc]^-^ |
| 35^a^ | 13.30 | Ginsenoside Rc | C_53_H_90_O_22,_ | [M-H+HCOOH]^-^ | 1123.5895 | 1123.5935 | 3.584 | 945.5285 [M-H-Araf]^-^, 783.5462 [M-H-Araf-Glc]^-^, 621.4621 [M-H-Araf-2Glc]^-^, 459.5153 [M-H-Araf-3Glc]^-^ |
| 36 | 13.46 | 20(*R*)-ginsenoside Rh1 | C_36_H_62_O_9_ | [M-H+HCOOH]^-^ | 683.4365 | 683.4383 | 2.635 | 637.4329 [M-H]^-^, 475.3799 [M-H-Glc]^-^ |
| 37 | 13.56 | Ginsenoside Ra2 | C_58_H_98_O_26_ | [M-H]^-^ | 1209.6263 | 1209.6268 | 0.472 | 1077.5461 [M-H-Xyl]^-^, 945.5256 [M-H-Xyl-Araf]^-^, 783.5811 [M-H-Xyl-Araf-Glc]^-^, 621.5166 [M-H-Xyl-Araf-2Glc]^-^, 459.5353 [M-H-Xyl-Araf-3Glc]^-^ |
| 38^a^ | 13.62 | Ginsenoside Rb2 | C_53_H_90_O_22_ | [M-H+HCOOH]^-^ | 1123.5895 | 1123.5920 | 2.279 | 945.5582 [M-H-Arap]^-^, 783.6059 [M-H-Arap-Glc]^-^, 621.5035 [M-H-Arap-2Glc]^-^, 459.4655 [M-H-Arap-3Glc]^-^ |
| 39 | 13.92 | Ginsenoside Rb3 | C_53_H_90_O_22_ | [M-H+HCOOH]^-^ | 1123.5895 | 1123.5928 | 2.928 | 945.5981 [M-H-Xyl]^-^, 783.4825 [M-H-Xyl-Glc]^-^, 621.3984 [M-H-Xyl-2Glc]^-^, 459.3667 [M-H-Xyl-3Glc]^-^ |

**Table 1** (*Continued*)

| No. | *t*_R_  (min) | Identification | Molecular  formula | Proposal ions | Theoretical  mass (*m/z*) | Experimental  mass (*m/z*) | Mass error (ppm) | MS^2^ fragment ions (*m/z*) | |
| --- | --- | --- | --- | --- | --- | --- | --- | --- | --- |
| 40 | 14.09 | Quinquenoside L1 | C_48_H_80_O_18_ | [M-H]^-^ | 943.5261 | 943.5269 | 0.814 | 781.4971 [M-H-Glc]^-^, 619.4375 [M-H-2Glc]^-^, 457.3902 [M-H-3Glc]^-^ | |
| 41 | 14.30 | Ginsenoside F1 | C_36_H_62_O_9_ | [M-H+HCOOH]^-^ | 683.4365 | 683.4387 | 3.176 | 637.3828 [M-H]^-^, 475.3951 [M-H-Glc]^-^ | |
| 42^a^ | 14.56 | Ginsenoside Rd | C_48_H_82_O_18_ | [M-H+HCOOH]^-^ | 991.5472 | 991.5497 | 2.480 | 783.5506 [M-H-Glc]^-^, 621.5385 [M-H-2Glc]^-^, 459.5683 [M-H-3Glc]^-^ | |
| 43 | 15.66 | Ginsenoside Rh14/Rh17 | C_42_H_70_O_13_ | [M-H+HCOOH]^-^ | 827.4787 | 827.4812 | 2.964 | 781.4617 [M-H]^-^, 635.3858 [M-H-Rha]^-^, 473.3018 [M-H-Rha-Glc]^-^ | |
| 44 | 16.61 | Ginsenoside Rg4/Rg6 | C_42_H_70_O_12_ | [M-H+HCOOH]^-^ | 811.4838 | 811.4858 | 2.399 | 619.5092 [M-H-Rha]^-^, 457.5426 [M-H-Rha-Glc]^-^ | |
| 45 | 16.90 | Ginsenoside Rg4/Rg6 | C_42_H_70_O_12_ | [M-H+HCOOH]^-^ | 811.4838 | 811.4862 | 2.855 | 619.5980 [M-H-Rha]^-^, 457.4846 [M-H-Rha-Glc]^-^ | |
| 46^a^ | 17.39 | Ginsenoside F2 | C_42_H_72_O_13_ | [M-H+HCOOH]^-^ | 829.4944 | 829.4960 | 1.968 | 783.5378 [M-H]^-^, 621.4547 [M-H-Glc]^-^, 459.4137 [M-H-2Glc]^-^ | |
| 47 | 17.87 | Ginsenoside Rh15 | C_42_H_70_O_13_ | [M-H+HCOOH]^-^ | 827.4787 | 827.4808 | 2.516 | 619.5326 [M-H-Glc]^-^, 457.3878 [M-H-2Glc]^-^ | |
| 48^a^ | 18.37 | 20(*S*)-ginsenoside Rg3 | C_42_H_72_O_13_ | [M-H+HCOOH]^-^ | 829.4944 | 829.4966 | 2.638 | 621.4397 [M-H-Glc]^-^, 459.4392 [M-H-2Glc]^-^ | |
| 49 | 18.56 | 20(*R*)-ginsenoside Rg3 | C_42_H_72_O_13_ | [M-H+HCOOH]^-^ | 829.4944 | 829.4961 | 2.048 | 621.3977 [M-H-Glc]^-^, 459.4046 [M-H-2Glc]^-^ | |
| 50 | 20.60 | Notoginsenoside T5 | C_41_H_68_O_12_ | [M-H+HCOOH]^-^ | 797.4682 | 797.4705 | 2.930 | 751.5647 [M-H]^-^, 619.4392 [M-H-Xyl]^-^, 457.5015 [M-H-Xyl-Glc]^-^ | |
| 51 | 21.10 | Ginsenoside Rg5/Rz1 | C_42_H_70_O_12_ | [M-H+HCOOH]^-^ | 811.4838 | 811.4855 | 2.017 | 603.3588 [M-H-Glc]^-^, 441.4138 [M-H-2Glc]^-^ | |
| 52 | 21.59 | Ginsenoside Rg5/Rz1 | C_42_H_70_O_12_ | [M-H+HCOOH]^-^ | 811.4838 | 811.4858 | 2.399 | 603.4473 [M-H-Glc]^-^, 441.3460 [M-H-2Glc]^-^ | |
| 53^a^ | 21.97 | Ginsenoside compound K | C_36_H_62_O_8_ | [M-H+HCOOH]^-^ | 667.4416 | 667.4434 | 2.675 | 621.4007 [M-H]^-^, 459.4694 [M-H-Glc]^-^ | |
| 54 | 22.39 | 20(*S*)-ginsenoside Rh2 | C_36_H_62_O_8_ | [M-H+HCOOH]^-^ | 667.4416 | 667.4435 | 2.855 | 621.4597 [M-H]^-^, 459.3947 [M-H-Glc]^-^ | |
| 55^a^ | 22.68 | 20(*R*)-ginsenoside Rh2 | C_36_H_62_O_8_ | [M-H+HCOOH]^-^ | 667.4416 | 667.4437 | 3.125 | 621.5268 [M-H]^-^, 459.5037 [M-H-Glc]^-^ | |
| 56 | 23.20 | Ginsenoside Rh16 | C_36_H_60_O_8_ | [M-H+HCOOH]^-^ | 665.4259 | 665.4282 | 3.360 | 619.3924 [M-H]^-^, 457.4629 [M-H-Glc]^-^ | |
| Glc: *β*-D-glucose, Arap: *α*-L-arabinose (pyranose), Araf: *α*-L-arabinose (furanose), Rha: *α*-L-rhamnose, Xyl: *β*-D-xylose.  ^a^ Identified with reference standard. | | | | | | | | |  |

## 2.2 Quantitative Analysis of GE

Nine ginsenosides were determined in GE sample on on the AB Sciex Triple Quad™ 4500 system (Applied Biosystems Inc.) using negative ion electrospray ionization and multiple reaction monitoring (MRM) mode. The reference substances of ginsenosides (Ginsenoside Re, Rg1, Rf, Rb1, Rc, 20(*S*)-Rg2, Rb2, 20(*S*)-Rh1, Rd) were purchased from Chengdu Must Biotechnology Co., Ltd. (Chengdu, China). Chromatographic separation was performed on an ACQUITY HSS T3 C18 UPLC column (100 mm × 2.1 mm i.d., 1.8 µm) at 30 °C. Acetonitrile (A) and 0.1% (*v/v*) formic acid in water (B) were as elution solvents at a flow rate of 0.2 mL/min and the gradient program was as follows: 0-3 min, 30%; 3-5 min, 30-40% A; 5-8 min, 40% A; 8-11 min, 40-70% A; 11-13 min, 70-100% A. The injection volume was 10 µL. The instrument was operated under the following setting parameters: source temperature, 500 °C; ion spray voltage, -4500 kV; curtain gas, 20 psi; nebulizing gas, 50 psi; heater gas, 50psi. The MRM transitions, collision energy and declustering potential of nine analytes were list in Table 2. AB SCIEX Analyst 1.6.3 software was used for data analysis.

The quantification method was validated in terms of linearity, lower limit of detection (LLOD) and quantification (LLOQ), precision, stability, and accuracy. All calibration curves were constructed by plotting the peak areas (*y*) of each extracting ion versus the concentrations (*x*, ng/mL) of the nine analytes. The LLOD and LLOQ were determined at a signal-to-noise of about 3 and 10, respectively. Intra- and inter-day variations were obtained to evaluate the precision of the developed assay. The stability test was performed by analyzing the GE sample solution at 0 h, 2 h, 4 h, 8 h, 12 h, 24 h, and 48 h. The recovery test was used to assess the accuracy of the method. A known amount of standard references was spiked into sample and analyzed using the above method. The represent chromatograms under the qualitative analysis condition were shown in Figure 3. The results of method validation were shown in Table 3-4.

The established method was applied for the quantitative evaluation of GE, and the contents of ginsenoside Re, Rg1, Rf, Rb1, Rc, 20(*S*)-Rg2, Rb2, 20(*S*)-Rh1, Rd were 14.46%, 3.49%, 2.02%, 12.78%, 13.02%, 1.00%, 7.52%, 0.27%, 3.64%, respectively.

**Table 2** The MRM parameters and contents of nine analytes in GE.

| Analyte | t*_R_* (min) | MRM transition (*m/z*) | Declustering potential (V) | Collision energy (V) |
| --- | --- | --- | --- | --- |
|  |  | Precursor ion→product ion |  |  |
| Re | 2.19 | 945.5 → 475.3 | -250 | -70 |
| Rg1 | 2.25 | 799.2 → 637.3 | -225 | -43 |
| Rf | 6.19 | 799.6 → 475.3 | -205 | -56 |
| Rb1 | 6.45 | 1107.5 → 621.2 | -280 | -75 |
| Rc | 6.69 | 1077.6 → 621.5 | -230 | -65 |
| 20(*S*)-Rg2 | 6.92 | 783.6 → 475.3 | -209 | -58 |
| Rb2 | 6.97 | 1077.6 → 783.4 | -230 | -65 |
| 20(*S*)-Rh1 | 7.13 | 637.5 → 475.5 | -190 | -39 |
| Rd | 8.05 | 945.3 → 783.4 | -250 | -51 |

**
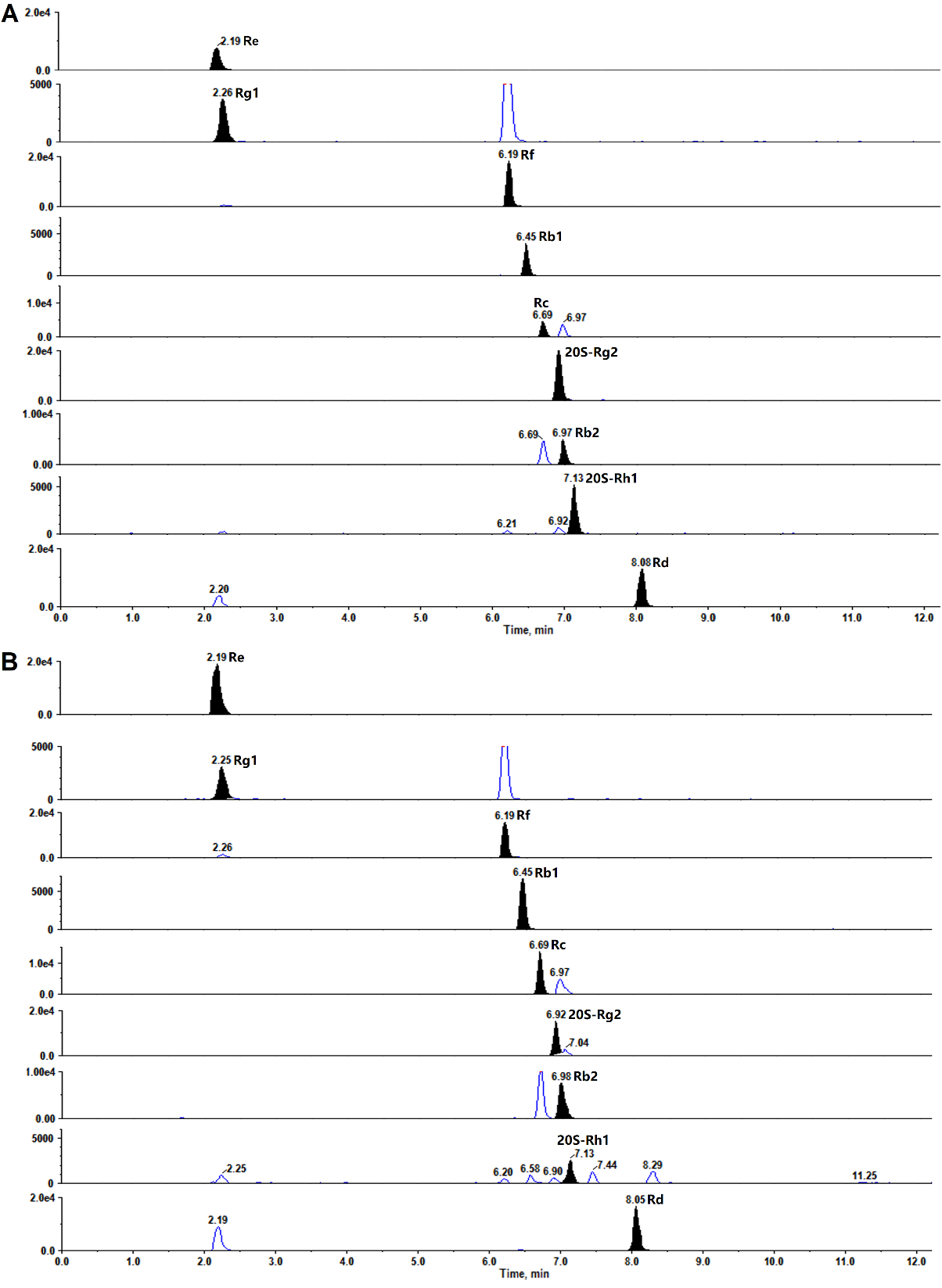
**

**Figure 3.** The typical MRM chromatograms of ginsenosides in the negative ion mode by UPLC-MS/MS. (A) mixed standards; (B) GE.

**Table 3** Linear calibration curves of nine ginsenosides.

| Analyte | Linear equation | *r*^2^ | Linear range (ng/mL) | LLOD (ng/mL) | LLOQ (ng/mL) |
| --- | --- | --- | --- | --- | --- |
| Re | *y* = 1602.8325 *x* + 1422.6277 | 0.9999 | 1-1000 | 0.33 | 1 |
| Rg1 | *y* = 766.9904 *x* + 829.5109 | 1.0000 | 5-1000 | 0.67 | 2 |
| Rf | *y* = 5127.7434 *x* + 10409.7779 | 0.9999 | 1-1000 | 0.33 | 1 |
| Rb1 | *y* = 423.9834 *x* + 680.2633 | 0.9999 | 2-1000 | 0.67 | 2 |
| Rc | *y* = 429.7690 *x* + 3814.9189 | 0.9986 | 1-1000 | 0.33 | 1 |
| 20(*S*)-Rg2 | *y* = 8473.5545 *x* - 1311.4268 | 0.9995 | 1-1000 | 0.33 | 1 |
| Rb2 | *y* = 661.6140 *x* + 3115.7056 | 0.9997 | 2-1000 | 0.67 | 2 |
| 20(*S*)-Rh1 | *y* = 618.5184 *x* + 4940.0059 | 0.9985 | 1-1000 | 0.33 | 1 |
| Rd | *y* = 1712.9776 *x* + 23623.1369 | 0.9982 | 1-1000 | 0.33 | 1 |
| F2 | *y* = 1780.9921 *x* + 15883.3918 | 0.9990 | 1-1000 | 0.33 | 1 |

**Table 4** Precision, stability, and accuracy of nine ginsenosides.

| Analyte | Precision (RSD, %) | | Stability  (RSD, %) | Recovery  (%) |
| --- | --- | --- | --- | --- |
|  | Intra-day | Inter-day |  |  |
| Re | 2.81 | 2.49 | 2.30 | 100.10 |
| Rg1 | 2.31 | 1.27 | 2.39 | 97.81 |
| Rf | 2.01 | 2.43 | 1.24 | 99.54 |
| Rb1 | 2.24 | 2.53 | 2.40 | 99.47 |
| Rc | 2.72 | 1.97 | 2.72 | 99.78 |
| 20(*S*)-Rg2 | 2.49 | 2.64 | 2.69 | 99.53 |
| Rb2 | 2.63 | 2.79 | 2.78 | 100.01 |
| 20(*S*)-Rh1 | 2.65 | 2.62 | 2.60 | 98.21 |
| Rd | 1.90 | 2.64 | 1.90 | 99.29 |
| F2 | 2.33 | 2.35 | 2.64 | 98.56 |

# 3 Supplementary Tables and Figures

## 3.1 Supplementary Tables

**Supplementary** **Table S1** Primers used in this study

| Name | Sequence (5'→3') |
| --- | --- |
| SREBP-1c Forward | GCACTGAGGCAAAGCTGAAT |
| SREBP-1c Reverse | CAGTGCGCAGACTTAGGTTC |
| FAS Forward | CCTTCATCGGGATGGAGTCT |
| FAS Reverse | TGATCTTCATGGTGCTGGGT |
| ACC-1 Forward | ACCCACTCCACTGTTTGTGA |
| ACC-1 Reverse | GGCCTTTGTGGGAACAAACT |
| CPT-1a Forward | ATAAGGATCTGGCGGAGTGG |
| CPT-1a Reverse | TCCATGGCAACCTCTGGATT |
| TNF-α Forward | CTGCACTTTGGAGTGATCGG |
| TNF-α Reverse | AGGGTTTGCTACAACATGGG |
| IL-1β Forward | AGCAACAAGTGGTGTTCTCC |
| IL-1β Reverse | CCAGCTGTAGAGTGGGCTTA |
| ZO-1 Forward | TGAAATCGCACAGTTTGGCA |
| ZO-1 Reverse | CAGCTGAAGGACTCACAGGA |
| Occludin Forward | CATGGCTGCTGCTGATGAAT |
| Occludin Reverse | ACAACTTGGCATCAGCCTTC |
| GAPDH Forward | CAGAACATCATCCCTGCATC |
| GAPDH Reverse | TACTTGGCAGGTTTCTCCAG |

## 3.2 Supplementary Figures


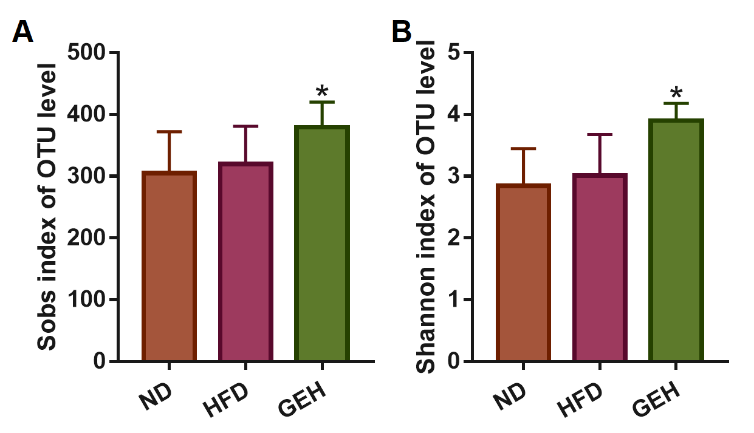


**Supplementary** **Figure S1.** Effects of different treatments on gut microbial composition. (A-B) Richness and diversity of microbial composition, estimated by Sobs index and Shannon index among the four groups. n = 8 per group. **p* < 0.05 vs. the HFD group.


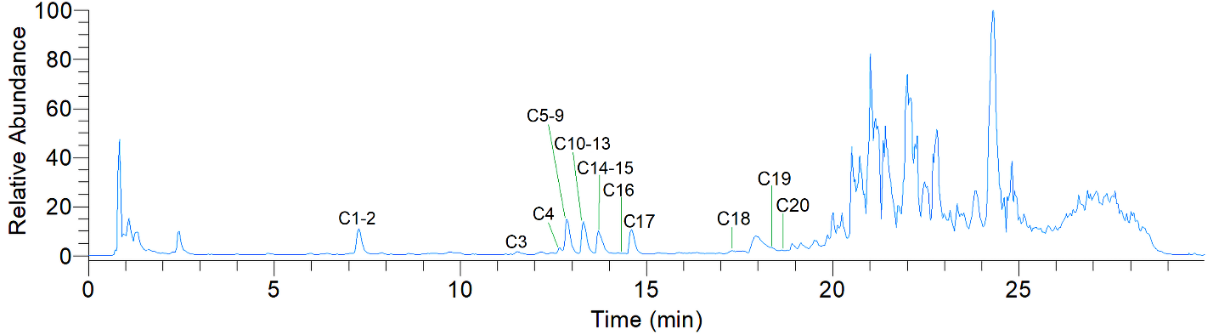


**Supplementary** **Figure S2.** Total ion current chromatograms of drug-containing plasma sample of mice after oral administration of GE in negative mode. The peak number corresponds to the compound number in the manuscript section “Network pharmacology analysis based on the components in the plasma”.


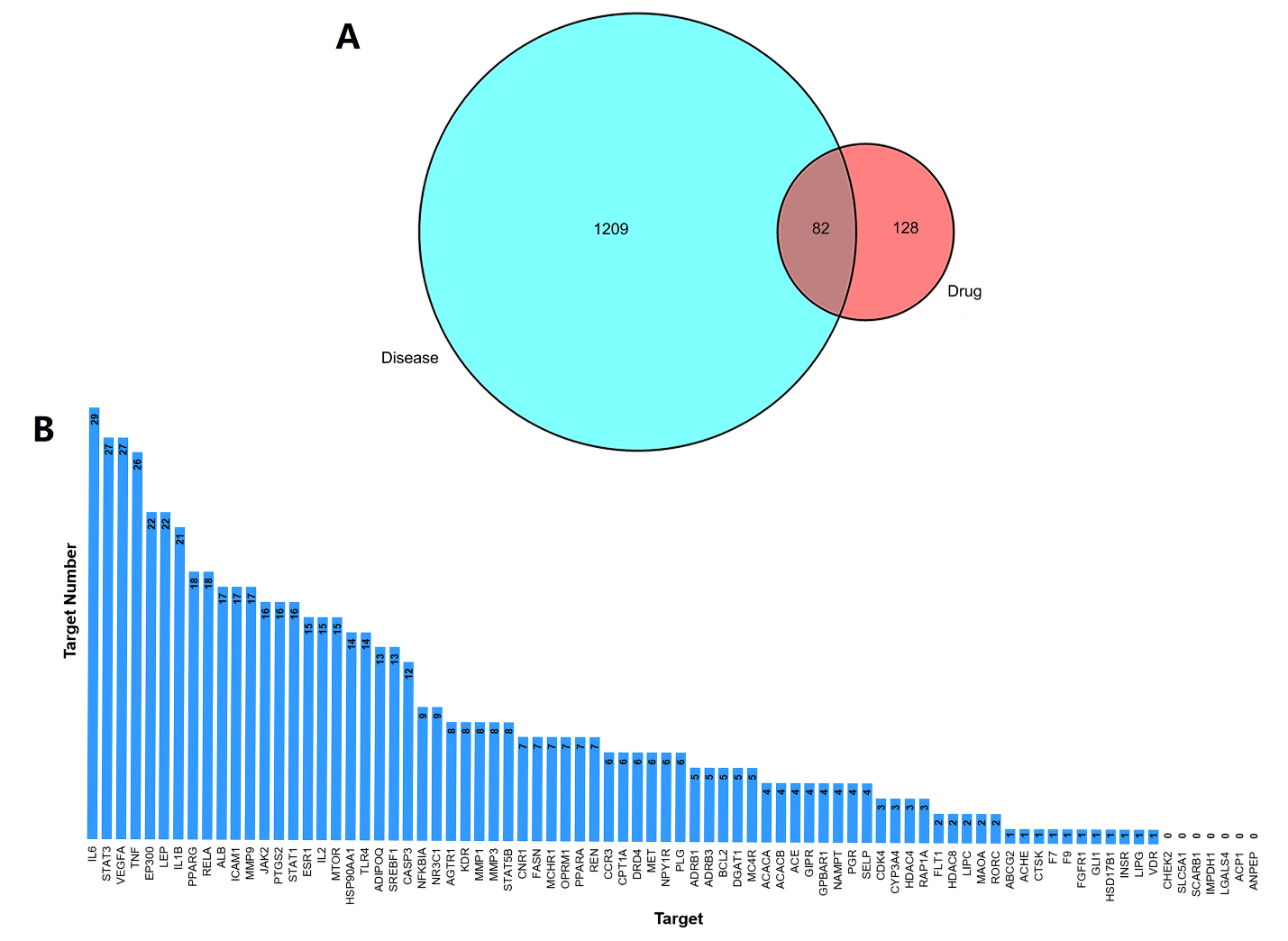


**Supplementary** **Figure S3.** Network pharmacology analysis of GE. (A) Venn diagram: to identified putative target genes by mapping drug targets to disease targets. (B) Putative targets of GE based on PPI network analysis.


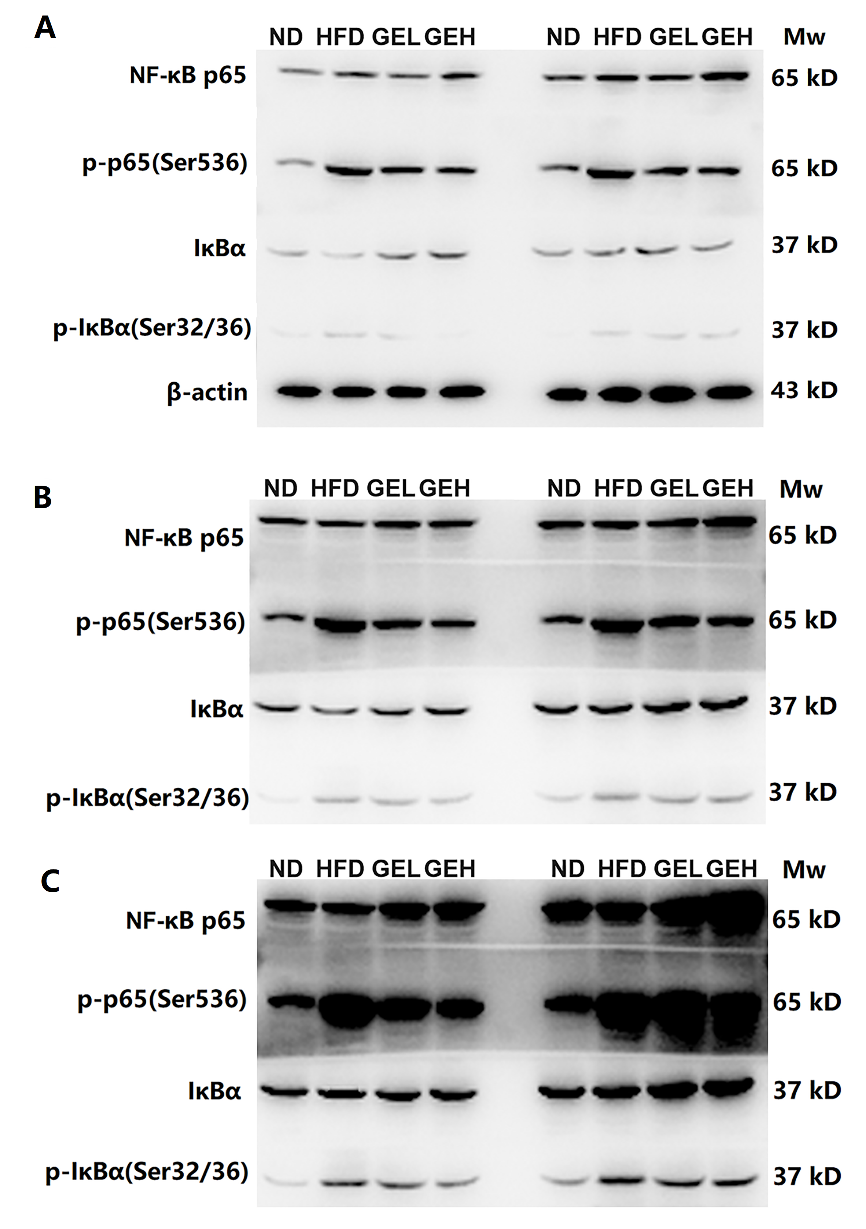


**Supplementary** **Figure S4.** The un-cropped images of the original western blots for Figure 7D. (A-C) The exposure time were 10 secs, 30 secs, 120 secs, respectively.

**Reference**

Li, S.L., Lai, S.F., Song, J.Z., Qiao, C.F., Liu, X., Zhou, Y., et al. (2010). Decocting-induced chemical transformations and global quality of Du-Shen-Tang, the decoction of ginseng evaluated by UPLC-Q-TOF-MS/MS based chemical profiling approach. *J Pharm Biomed Anal* 53**,** 946-957. doi: 10.1016/j.jpba.2010.07.001.

Qiu, S., Yang, W.Z., Shi, X.J., Yao, C.L., Yang, M., Liu, X., et al. (2015). A green protocol for efficient discovery of novel natural compounds: characterization of new ginsenosides from the stems and leaves of Panax ginseng as a case study. *Anal Chim Acta* 893**,** 65-76. doi: 10.1016/j.aca.2015.08.048.

Xie, Y.Y., Luo, D., Cheng, Y.J., Ma, J.F., Wang, Y.M., Liang, Q.L., et al. (2012). Steaming-induced chemical transformations and holistic quality assessment of red ginseng derived from Panax ginseng by means of HPLC-ESI-MS/MS(n)-based multicomponent quantification fingerprint. *J Agric Food Chem* 60**,** 8213-8224. doi: 10.1021/jf301116x.
